# Four Novel d^10^ Metal-Organic Frameworks Incorporating Amino-Functionalized Carboxylate Ligands: Synthesis, Structures, and Fluorescence Properties

**DOI:** 10.3389/fchem.2021.708314

**Published:** 2021-08-30

**Authors:** Wang Xie, Jie Wu, Xiaochun Hang, Honghai Zhang, Kang shen, Zhoulu Wang

**Affiliations:** ^1^ Key Laboratory of Flexible Electronics & Institute of Advanced Materials, Jiangsu National Synergistic Innovation Center for Advanced Materials, School of Energy Science and Engineering, Nanjing Tech University, Nanjing, China; ^2^ State Key Laboratory of Coordination Chemistry, Nanjing University, Nanjing, China

**Keywords:** metal-organic frameworks, d^10^ -metal ions, amino groups, fluorescence, detection

## Abstract

By employment of amino-functionalized dicarboxylate ligands to react with d^10^ metal ions, four novel metal-organic frameworks (MOFs) were obtained with the formula of {[Cd(BCPAB)(*μ*_2_-H_2_O)]}_*n*_ (1), {[Cd(BDAB)]∙2H_2_O∙DMF}_*n*_ (2), {[Zn(BDAB)(BPD)_0.5_(H_2_O)]∙2H_2_O}_*n*_ (3) and {[Zn(BDAB)(DBPB)_0.5_(H_2_O)]∙2H_2_O}_*n*_ (4) (H_2_BCPAB = 2,5-bis(p-carbonylphenyl)-1-aminobenzene; H_2_BDAB = 1,2-diamino-3,6-bis(4-carboxyphenyl)benzene); BPD = (4,4′-bipyridine); DBPB = (*E,E-*2,5-dimethoxy-1,4-bis-[2-pyridin-vinyl]-benzene; DMF = *N*,*N*-dimethylformamide). Complex 1 is a three-dimensional (3D) framework bearing *seh*-3,5-*Pbca* nets with point symbol of {4.6^2^}{4.6^7^.8^2^}. Complex 2 exhibits a 4,4-connected new topology that has never been reported before with point symbol of {4^2^.8^4^}. Complex 3 and 4 are quite similar in structure and both have 3D supramolecular frameworks formed by 6-fold and 8-fold interpenetrated 2D coordination layers. The structures of these complexes were characterized by single crystal X-ray diffraction (SC-XRD), thermal gravimetric analysis (TGA) and powder X-ray diffraction (PXRD) measurements. In addition, the fluorescence properties and the sensing capability of 2–4 were investigated as well and the results indicated that complex 2 could function as sensor for Cu^2+^ and complex 3 could detect Cu^2+^ and Ag^+^ *via* quenching effect.

## Introduction

Metal-organic frameworks (MOFs), which are formed by coordination bonds between metal nodes and organic linkers (Tranchemontagne et al., 2009), have been one of the most rapidly developing areas of material science, not only because of the tunable porosity, controlled structure, and readily chemical functionalization of these materials, but also because of their wide potential applications such as heterogeneous catalysis, gas adsorption and storage, chemical sensing and explosive detection, drug delivery, and optoelectronics. (Barea et al., 2014; Canivet et al., 2014; Dhakshinamoorthy and Garcia, 2014; He et al., 2014; Hu et al., 2014; Liu et al., 2014; Van de Voorde et al., 2014; Silva et al., 2015; Zhu et al., 2015; Sheberla et al., 2017; Li et al., 2018; Park et al., 2018; Prasad et al., 2018; Wang et al., 2018; Cao et al., 2019; Mallick et al., 2019) For example, as a kind of new absorbent materials, quantities of MOFs have been widely investigated in the capture and separation of various gases, such as CO_2_, SO_2_, H_2_S, NH_3_, hydrocarbons and so on. (Li et al., 2009; Peng et al., 2013; Zhang et al., 2014; Trickett et al., 2017; Zárate et al., 2019a; Zárate et al., 2019b; Tchalala et al., 2019; Wang et al., 2020; Han et al., 2021) Varieties of MOFs have also been explored as luminescent materials in different fields, for example, sensing, nonlinear optical materials, OLED, and so forth. (Lustig et al., 2017; Medishetty et al., 2017; Gutiérrez et al., 2018) Although many MOFs have exhibited relatively superior performance, the majority of them do not meet the requirements of practical applications. In order to further improve the properties of MOFs, some strategies have been proposed in previous reports and the introduction of substituent groups into the organic ligands has been proved one of the most effective manners. Among various substituent groups, the influence of amino groups on the structures and properties of MOFs has been intensively studied because amino groups could coordinate with metal ions and form hydrogen bonds with guest molecules, which thus may strengthen some performance or even endow MOFs more functionalities. For instance, Hu et al. demonstrated that the supramolecular interactions of C-H···O, C···O, and O···O could distinctly enhance the adsorption capacity for CO_2_. (Hu et al., 2015) Dong and co-workers found that the introduction of amino groups to UiO-66 could provide sensing capability towards lysine and arginine *via* fluorescence turn-on effect. (Dong et al., 2020)

In consideration of the positive effect of amino groups on the properties of MOFs, we employed amino-functionalized dicarboxylate ligands to construct MOFs in this work. For this purpose, ligands 2,5-bis(p-carbonylphenyl)-1-aminobenzene (H_2_BCPAB) and 1,2-diamino-3,6-bis(4-carboxyphenyl)benzene) (H_2_BDAB) were synthesized to react with d^10^ metal ions Zn^2+^ and Cd^2+^ in the absence and presence of auxiliary ligands and four novel MOFs with the formula of {[Cd(BCPAB)(*μ*_2_-H_2_O)]}_*n*_ (1), {[Cd(BDAB)]∙2H_2_O∙DMF}_*n*_ (2), {[Zn(BDAB)(BPD)_0.5_(H_2_O)]∙2H_2_O}_*n*_ (3) and {[Zn(BDAB)(DBPB)_0.5_(H_2_O)]∙2H_2_O}_*n*_ (4) (BPD = (4,4′-bipyridine); DBPB = (*E,E-*2,5-dimethoxy-1,4-bis-[2-pyridin-vinyl]-benzene; DMF = N,N-dimethylformamide) were obtained successfully. Their structure was determined and characterized by SC-XRD, TGA, and PXRD measurements. Besides, the fluorescence properties and the sensing capability of 2–4 were investigated as well, and the sensing experiments indicated that complex 2 could function as a sensor for Cu^2+^ and complex 3 could detect Cu^2+^ and Ag^+^ *via* quenching effect.

## Materials and Methods

**SCHEME 1:**
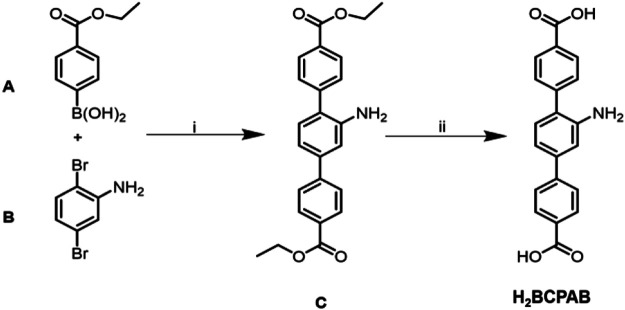
Synthesis and structures of ligand H_2_BCPAB. **(A)** Pd(PPh_3_)_4_, CsF, THF, N_2_; **(B)** KOH, THF/H_2_O.

**SCHEME 2:**
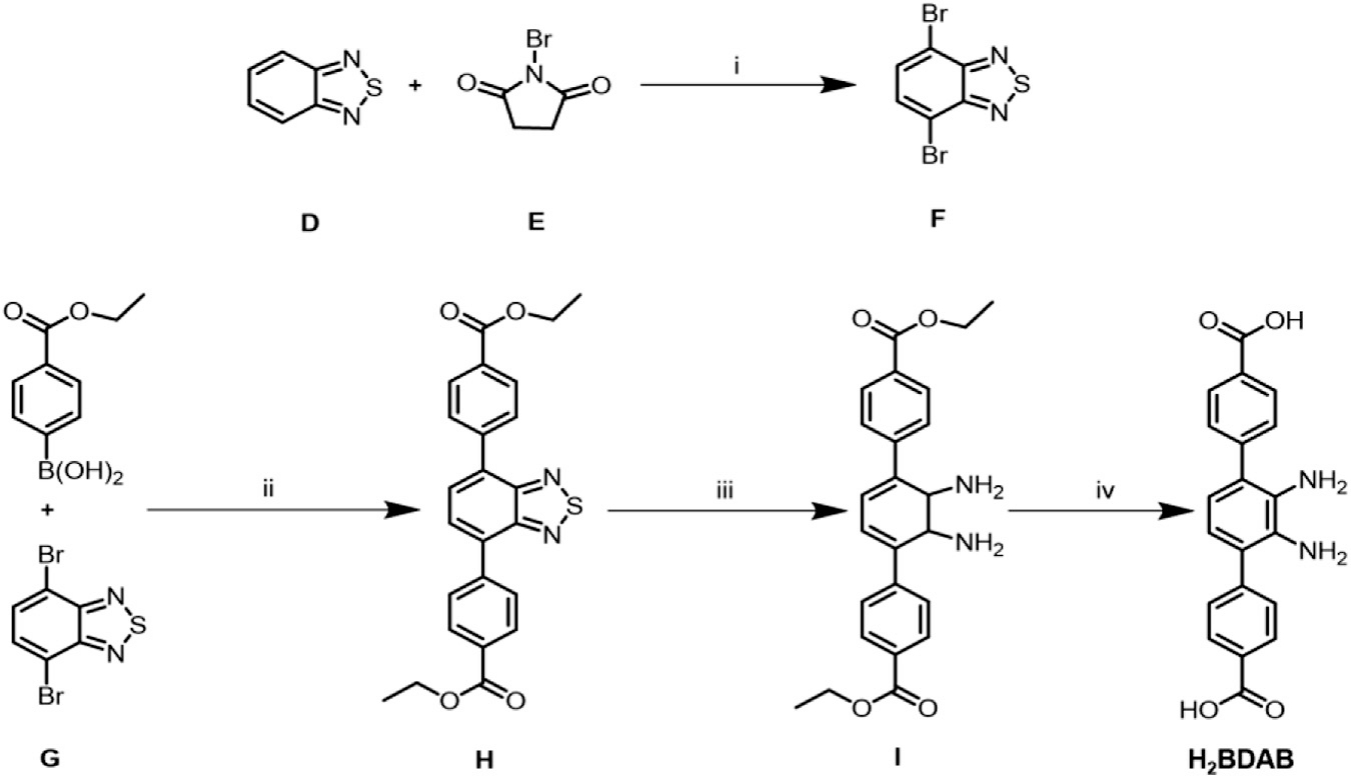
Synthesis and structures of ligand H_2_BDAB. **(A)** H_2_SO_4_ 98%; **(B)** Pd(PPh_3_)_4_, Cs_2_CO_3_, DMF/Toluene, N_2_; **(C)** NaBH4, CoCl_2_˖H_2_O; **(D)** KOH, THF/H_2_O.

**SCHEME 3:**
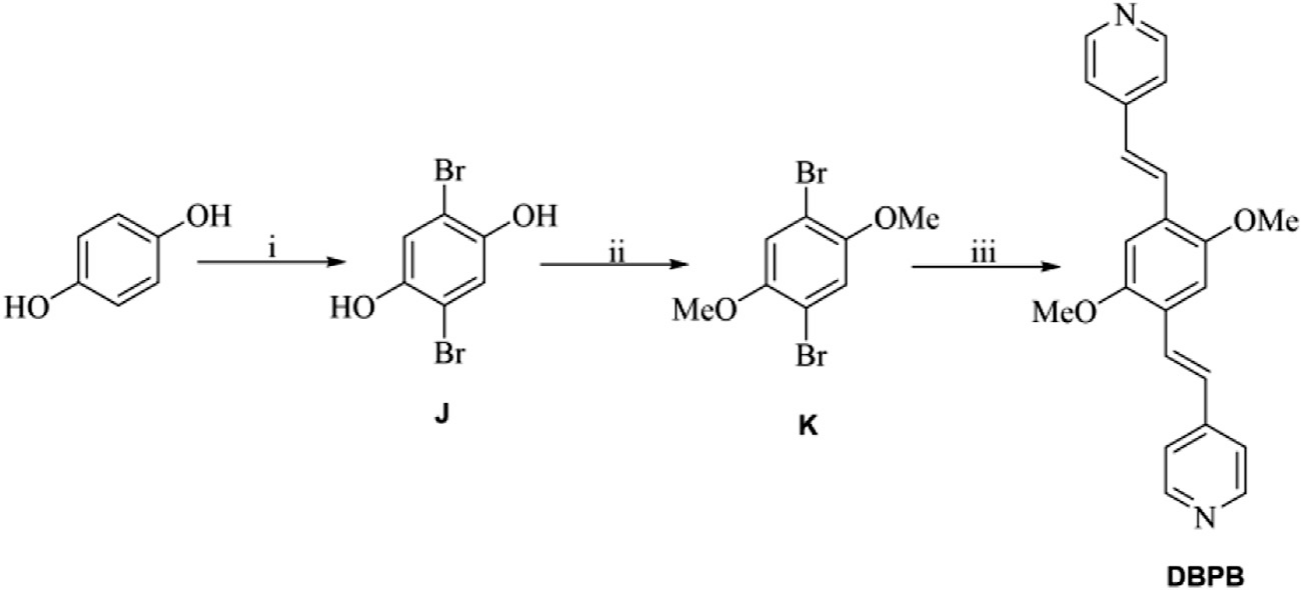
**(A)** Br_2_, AcOH; **(B)** K_2_CO_3_, acetone, CH_3_Br/(CH_3_)_2_SO_4_; **(C)** 4-vinylpyridine, Pd(PPh_3_)_4_, tris(2-methylphenyl)phosphine, Et_3_N, CH_3_CN.

All chemicals and solvents except the organic ligands H_2_BCPAB, H_2_BDAB, and DBPB were of reagent-grade quality from commercial sources and were used without further purification. The as-synthesized complexes were characterized by thermogravimetric analysis (TGA) on a Perkin Elmer thermogravimetric analyzer Pyris 1 TGA up to 500°C using a heating rate of 10°C min^−1^ under a N_2_ atmosphere. Powder X-ray diffraction (PXRD) measurements were performed on a Bruker D8 Advance X-ray diffractometer using Cu-K α radiation (1.5418 Å), and the X-ray tube was operated at 40 kV and 40 mA. The gas sorption isotherms were measured by using a Micromeritics ASAP 2020M + C surface area analyzer. Fluorescence spectra were recorded on a PerkinElmer LS-55 fluorescence spectrophotometer. Organic ligands H_2_BCPAB (Scheme 1), H_2_BDAB (Scheme 2) and DBPB (Scheme 3) were synthesized by previously reported procedures. (Nagarkar et al., 2015; Shen et al., 2016; Dutta et al., 2018)

### Synthesis of 2,5-bis(p-ethoxycarbonylphenyl)-1-aminobenzene (C)

2,5-bis(p-ethoxycarbonylphenyl)-1-aminobenzene (**C**) was synthesized by previously reported procedures. To a 1,000 ml round-bottom flask was added with 2, 5-dibromoaniline (1.2 g, 5 mmol), *p*-ethoxycarbonylphenylboronoc acid (2.9 g, 15 mmol), Pd(PPh_3_)_4_ (0.58 g, 0.5 mmol), CsF (3.6 g, 24 mmol) and tetrahydrofuran (75 ml). The mixture solution was bubbled with N_2_ for more than 10 min and refluxed for 3 days. TLC (hexane : ethyl acetate = 6:1) showed that the reaction has finished. After cooling to room temperature, water was added onto the reaction mixture and then extracted with ethyl acetate (30 ml × 3). The combined organic solution was dried with anhydrous MgSO_4_ and concentrated in vacuum. The crude residues were purified by column flash chromatography with the eluant (hexane:ethyl acetate = 30:1) to give a light yellow solid as target product (1.35 g, yield: 67.3%).

### Synthesis of 2,5-Bis (p-carbonylphenyl)-1-Aminobenzene (H_2_BCPAB).

Compound **C** (1.35 g, 3.4 mmol), KOH (6.24 g, 111 mmol), tetrahydrofuran (THF, 40 ml) and water (100 ml) were added to a 1 L round-bottom flask. The mixture solution was bubbled with N_2_ for more than 10 min and stirred at 50 C for 12 h. After removing THF in vacuum, the residue was added with water and then acidified with diluted HCl (1 M) until no precipitate formed. The atrovirens powder was collected by filtration as target product (0.88 g, yield: 72%). LC-MS (M + H)^+^_found_ = 334.12.

### Synthesis of 4,7-Dibromobenzo[c] (Tranchemontagne et al., 2009; Silva et al., 2015; Wang et al., 2018)Thiadiazole (F)

2,1,3-Benzothiadiazole (**D**) (0.5 g, 3.68 mmol), N-bromosuccinimide (NBS, 1.35 g, 7.61 mmol) and H_2_SO_4_ (98%, 5 ml) were added to a 25 ml round round-bottom flask. The reaction mixture was stirred at 60°C for 3 h. After cooling to room temperature, the solution was added with distilled water (25 ml) dropwise in an ice bath. The white desired solid was collected by filtration (1.06 g, yield: 97%).

### Synthesis of 4,7-bis(p-ethoxycarbonylphenyl)-2,1,3-benzothiodiazole (H)

Compound **F** (2.49 g, 10 mmol), *p*-ethoxycarbonylphenylboronoc acid (5.82 g, 30 mmol), Pd(PPh_3_)_4_ (1.16 g , 1 mmol), Cs_2_CO_3_ (8.15 g , 25 mmol), N,N-dimethylformamide (DMF, 100 ml), and toluene (100 ml) were added to a 500 ml round-bottom flask. The reaction solution was bubbled with N_2_ for more than 10 min and refluxed at 110°C for 24 h. TLC (hexane:ethyl acetate = 8:1) showed that the reaction has finished. The reaction solution was added with water and extracted with ethyl acetate (20 ml × 3). The combined organic solution was dried with anhydrous MgSO_4_ and concentrated in vacuum. The crude product was purified by column flash chromatography with the eluant (hexane:ethyl acetate = 40:1) to give an orange solid as the target product (2.25 g, yield: 49%).

### Synthesis of 3,6-bis(p-ethoxycarbonylphenyl)-1,2-diaminobenzene (I)

To a solution of **H** (1,296 mg, 3 mmol) in EtOH/THF (3:1, EtOH = ethyl alcohol) was added sodium borohydride (0.46 g, 12 mmol) and CoCl_2_·6H_2_O (29 mg, 0.12 mmol). The reaction solution was bubbled with N_2_ for more than 10 min and refluxed for 3 h. After removal of EtOH and THF, the residues were added with water and extracted with ethyl acetate (20 ml × 3). The combined organic phase was dried with anhydrous MgSO_4_ and concentrated in vacuum. The obtained solid was purified by column flash chromatography with the eluant (hexane:ethyl acetate = 20:1) to give a grey solid as the target product (0.92 mg, yield: 74.3%) . LC-MS (M + H)^+^_found_ = 405.30.

### Synthesis of 3,6-bis(p-carbonylphenyl)-1, 2-Diaminobenzene

Compound **I** (0.90 g, 2.22 mmol), KOH (4.09 g, 73 mmol), THF (20 ml), and water (60 ml) were added to a 500 ml round-bottom flask. The reaction solution was bubbled with N_2_ for more than 10 min and stirred at 60 C for 24 h. After removing THF in vacuum, the mixture was added with water and then acidified with diluted HCl (1 M) until no precipitate formed. The yellow powder was collected by filtration as target product (0.63 mg, 82% yield). LC-MS (M + H)^+^_found_ = 349.23.

### Synthesis of {[Cd(BCPAB) (*μ*_2_-H_2_O)]}_*n*_ (1)

A mixture of H_2_BCPAB (6.7 mg, 0.02 mmol), Cd(NO_3_)_2_∙4H_2_O (31 mg, 0.1 mmol), DMA (3.5 ml), H_2_O (3 ml) was placed in a 25 ml glass vial and heated at 95°C for 4 days. The resultant plate crystals were washed with fresh DMA and collected. Yield: 72% (based on H_2_BCPAB).

### Synthesis of [Cd(BDAB)]∙2H_2_O∙DMF}_*n*_ (2)

A mixture of H_2_BDAB (7 mg, 0.02 mmol), Cd(NO_3_)_2_∙4H_2_O (31 mg, 0.1 mmol), DMF (2 ml), H_2_O (4 ml) was placed in a 25 ml glass vial and heated at 95°C for 4 days. The resultant plate crystals were washed with fresh DMA and collected. Yield: 93% (based on H_2_BDAB).

### Synthesis of {[Zn(BDAB)(BPD)_0.5_(H_2_O)]∙2H_2_O}_*n*_ (3)

A mixture of H_2_BDAB (7 mg, 0.02 mmol), BPD (1.5 mg, 0.01 mmol), Zn(NO_3_)_2_∙6H_2_O (30 mg, 0.1 mmol), DMF (3.5 ml), H_2_O (2.5 ml) was placed in a 25 ml glass vial and heated at 100°C for 3 days. The resultant red featheriness crystals were washed with fresh DMF and collected. Yield: 36% (based on H_2_BDAB).

### Synthesis of {[Zn(BDAB)(DBPB)_0.5_(H_2_O)]∙2H_2_O}_*n*_ (4)

A mixture of H_2_BDAB (7 mg, 0.02 mmol), DBPB (3.5 mg, 0.01 mmol), Zn(NO_3_)_2_∙6H_2_O (30 mg, 0.1 mmol), DMF (3.5 ml), and H_2_O (1.5 ml) was placed in a 25 ml glass vial and heated at 105°C for 3 days. The resultant red featheriness crystals were washed with fresh DMF and collected. Yield: 59% (based on H_2_BDAB).

## Results and Discussion

### Description of the Crystal Structure of {[Cd(BCPAB)(*μ*_2_-H_2_O)]}_*n*_ (1)

**FIGURE 1:**
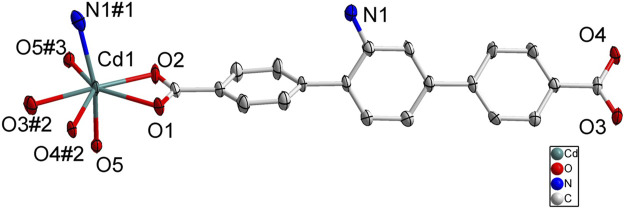
Coordination environment of Cd(II) cation in 1 with ellipsoids drawn at 50% probability level. The hydrogen atoms are omitted for clarity. Symmetry codes: #1 1-*x*, -1/2 + *y*, 3/2-*z*; #2 *x*, 3/2-*y*, -1/2 + *z*; #3 1/2-*x*, -1/2 + *y*, *z*.

**FIGURE 2:**
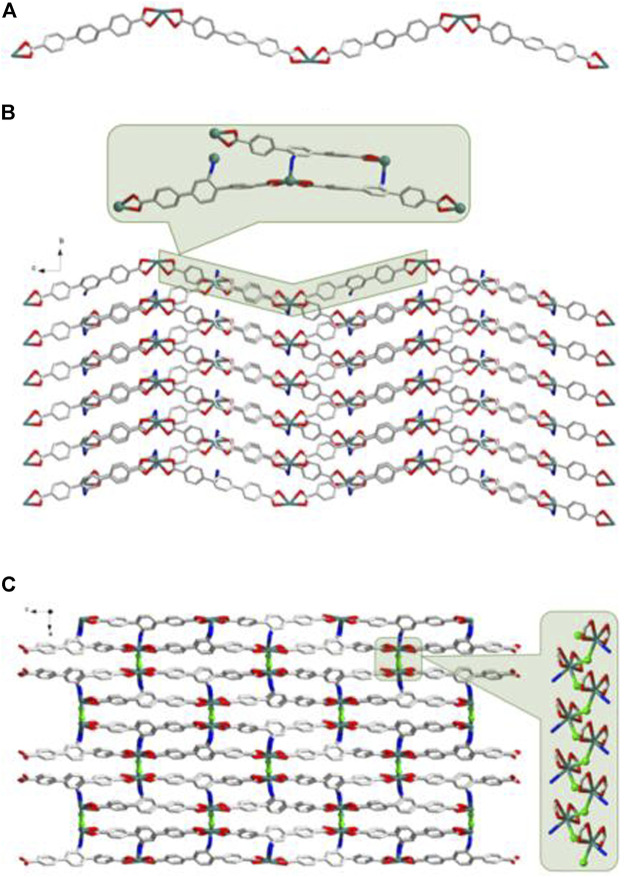
**(A)** The 1D Cd^2+^-BCPAB^2-^ chain. Amino groups were omitted for clarity. **(B)** The 2D coordination layers constructed from the Cd^2+^-BCPAB^2-^ chains *via* the bonds between amino groups and Cd^2+^ cations. **(C)** The 3D frameworks formed by the coordination between the bridged *μ*_2_-H_2_O molecules (green) with Cd^2+^ cations from different 2D Cd^2+^-BCPAB^2-^ layers and the 1D chains formed by Cd^2+^ cations and *μ*_2_-H_2_O molecules. Description of the crystal structure of [Cd(BDAB)]∙2H_2_O∙DMF}_*n*_ (2).

SC-XRD analysis revealed that complex **1** was crystallized in the orthorhombic system with a space group of *Pbca* and each asymmetric unit consisted of one Cd(II) metal center, one BCPAB^2−^ ligand and one water molecule. As shown in Figure 1, atom Cd1 adopted a distorted pentagonal bipyramid coordination geometry to coordinate with four carboxylate oxygen atoms (O1, O2, O3#2, O4#2) from two neighboring BCPAB^2−^ ligands, two coordinated water molecules (O5, O5#3) and one nitrogen atom (N1#1) from the amino group of BCPAB^2−^ ligand. The Cd-O bond lengths were in the range of 2.281–2.448 Å and the Cd-N bond length was 2.425 Å, which are comparable to the previous Cd-based coordination complexes. (Spek, 1998) Further structural analysis revealed that each carboxylate group of BCPAB^2−^ was bound to one Cd^2+^ ion and each Cd^2+^ ion coordinated with two carboxylate groups from two adjacent BCPAB^2−^ ligands, which thus resulted in the formation of one-dimensional (1D) coordination chains (Figure 2A). Furthermore, the coordination bonds between amino groups of the BCPAB^2−^ ligands and Cd^2+^ ions joined the 1D Cd^2+^-BCPAB^2−^ chains together to afford two-dimensional (2D) coordination layers (Figure 2B). On the other hand, each water molecule linked two Cd^2+^ ions to give 1D Cd-O coordination and thus the 2D Cd^2+^-BCPAB^2-^ layers were connected by the *μ*_2_-H_2_O molecules into the final 3D frameworks (Figure 2C; Supplementary Figure S1A). From the viewpoint of topology, the seven coordination secondary building unit and the BCPAB^2−^ ligand can be regarded as a 5-connected and 3-connected node, respectively, and the structure of 1 could be represented as *seh*-3, 5-*Pbca* nets with point symbol of {4.6^2^}{4.6^7^.8^2^} (Supplementary Figure. S1B).

**FIGURE 3:**
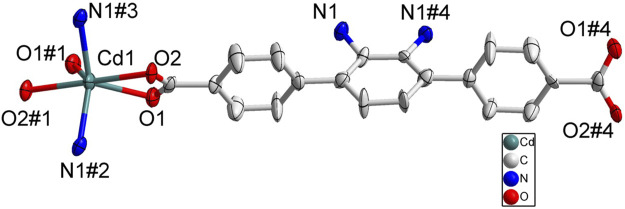
Coordination environment of Cd(II) cation in 2 with ellipsoids drawn at 50% probability level. The hydrogen atoms are omitted for clarity. Symmetry codes: #1 1/2-*x*, 1/2-*y*, -1/2-*z*; #2 -1/2 + *x*, 1/2 + *y*, -1/2 + *z*; #3 1-*x*, -*y*, -*z*.

**FIGURE 4:**
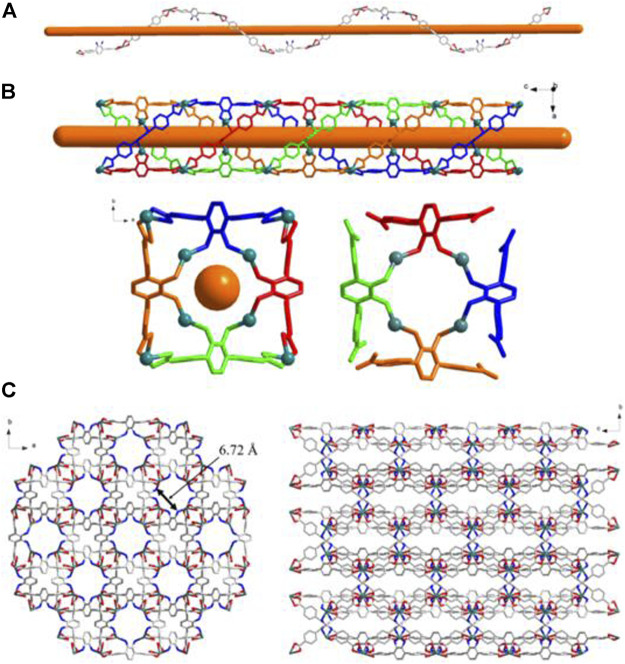
**(A)** The 1D helical coordination chain in 2. **(B)** The nanotube along b-axis and c-axis constructed from the coordination bonds between the amino groups from four different helical chains and Cd^2+^ cations. **(C)** Views of Twenty-membered-ring. (c) 3D frameworks of 2 along c and a axis.

According to SC-XRD measurements, complex 2 was crystallized in the tetragonal *P*4/*nnc* space group and each asymmetric unit contained half one Cd^2+^ and half one BDAB^2−^ ligand. As depicted in Figure 3, atom Cd1 was six-coordinated in a disordered octahedral coordination geometry with four carboxylate oxygen atoms (O1, O2, O1#1, O2#1) form two neighboring BDAB^2−^ ligands and two amino groups (N1#2, N1#3) from another two adjacent BDAB^2−^ ligands. Each Cd^2+^ ion connected two carboxylate groups from different BDAB^2−^ ligands to form 1D helical coordination chains (Figure 4A). Further inspection into the structure found that every four helical chains could assemble into a coordination nanotubular structure with the help of the binding between Cd^2+^ atoms and amino groups of BDAB^2−^ ligands on the chains (Figure 4B). The Cd^2+^ ions in the structure of nanotube were only coordinated with carboxylate groups or amino groups and thus they could bind to the amino groups or carboxylate groups from neighboring nanotubes, which then resulted in the formation of the final 3D coordination frameworks with 1D channels running along c-axis. From the point of topological view, because one Cd^2+^ cation connects four BDAB^2−^ ligands and one BDAB^2−^ ligand links four Cd^2+^ cations, the central Cd^2+^ cation and BDAB^2−^ ligand can both be treated as 4-connected nodes. Thence, the network of 2 can be represented with the point symbol is {4^2^.8^4^} calculated by TOPOS software (Supplementary Figure S2). The total solvent cavity volume of 2 is 35.0% per unit cell calculated by PLATON. (Zou et al., 2010)

**TABLE 1:** Crystal data and structural refinements parameters of 1–4.

Complex	1	2	3	4
Empirical formula	C_20_H_13_CdNO_5_	C_20_H_26_CdN_2_O_10_	C_25_H_24_ZnN_3_O_7_	C_31_H_30_ZnN_3_O_8_
Formula weight	459.72	530.81	543.86	637.97
Crystal system	Orthorhombic	Tetragonal	Monoclinic	Monoclinic
Space group	*Pbca*	*P*4*/nnc*	*C*2/*c*	*C*12/*c*1
*a* / Å	15.3465(4)	17.821	28.457(2)	36.105(2)
*b* / Å	5.6569(2)	17.821	6.2499(4)	5.9981(3)
*c* / Å	37.6515(11)	15.522	25.4312(18)	30.9842(16)
*α* / °	90.00	90.00	90.00	90.00
*β/* °	90.00	90.00	109.228(2)	105.301(4)
*γ*/ °	90.00	90.00	90.00	90.00
*V* / Å^3^	3268.66(17)	4929.5	4270.6(5)	6472.2(6)
*Z*	8	8	2	2
*D*_*calcd*_ / g cm^−3^	1.868	1.236	2.192	2.225
*μ* / mm^−1^	1.371	4.950	7.213	7.323
*F*(000)	1824	1824	2704	4160
*θ* min-max / °	1.082, 25.355	5.825, 53.987	1.696, 25.404	2.017, 27.586
Tot., uniq. data	17777, 5640	27501, 2280	11851, 3752	28571, 7474
*R*(int)	0.0822	0.0493	0.0433	0.0482
Nref, Npar	2953,244	2233, 111	3752, 315	7474, 380
*R*1, *wR*2 [I > 2*σ*(I)]	0.0653, 0.1118	0.1211, 0.2673	0.0687, 0.2014	0.0545, 0.1613
GOF on F^2^	1.069	1.120	1.175	1.038
Min. and max resd dens (e·Å^−3^)	−0.992, 1.442	−1.985, 3.493	−1.431, 0.687	−0.813, 0.852

R1 = *Σ*||Fo|-|Fc||/|*Σ*|Fo|; *w*R2 = {*Σ*[w(Fo2-Fc2)2]/*Σ*[*w*(Fo2)2]}1/2; where *w* = 1/[*σ*2(Fo2)+(aP)2 + bP],P=(Fo2+2Fc2)/3.

### Description of the Crystal Structure of {[Zn(BDAB)(BPD)_0.5_(H_2_O)]∙2H_2_O}_*n*_ (3) and {[Zn(BDAB)(DBPB)_0.5_(H_2_O)]∙2H_2_O}_*n*_ (4)

**FIGURE 5:**
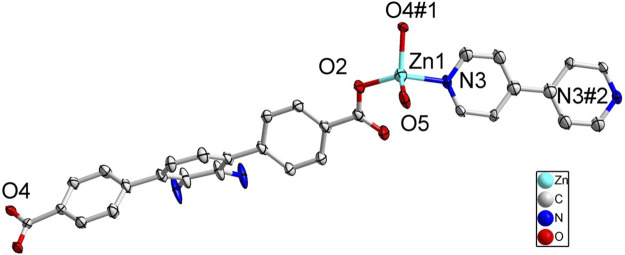
Coordination environment of Zn(II) cation in 3 with ellipsoids drawn at 50% probability level. The hydrogen atoms are omitted for clarity. Symmetry codes: #1 1/2 + *x*, 1/2-*y*, 1/2 + *z*; #2 3/2-*x*, 7/2-*y*, 2-*y*.

**FIGURE 6:**
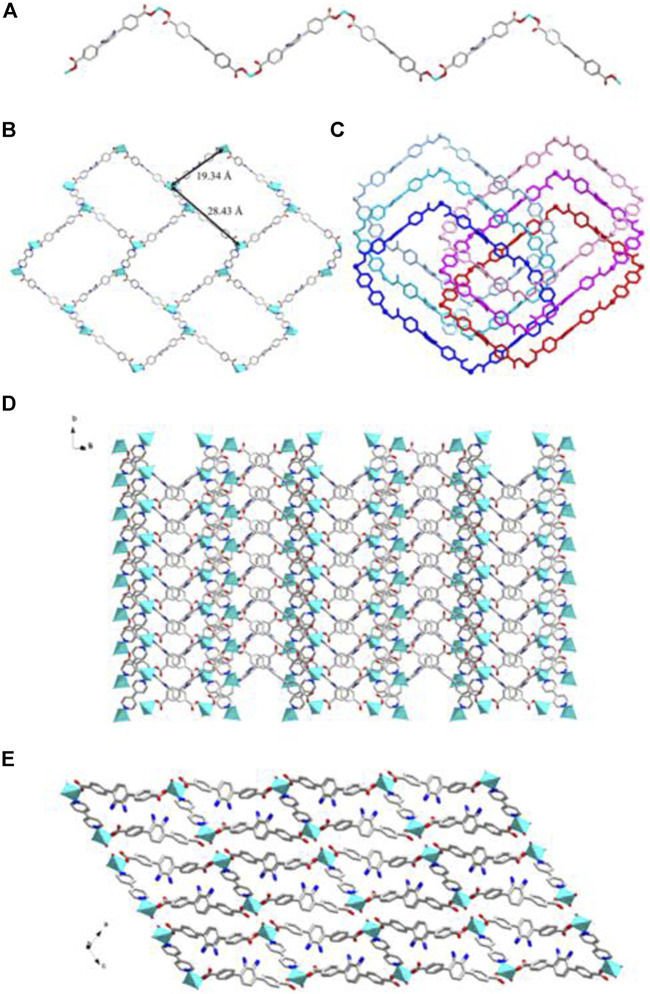
**(A)** The 1D BDAB^2-^-Zn^2+^ chains in 3. **(B)** The 2D BDAB^2-^-Zn^2+^-BPD coordination networks. **(C)** The interlaced mode of the adjacent BDAB^2-^-Zn^2+^-BPD networks. **(D)** The 2D interpenetrated BDAB^2-^-Zn^2+^-BPD layers. **(E)** The final 3D supramolecular architecture of **3**.

According to the results of SC-XRD measurements, complexes 3 and 4 were both crystallized in the monoclinic *C*2/*c* space group and shared a similar framework structure. Each asymmetric unit of 3 consisted of one Zn^2+^ cation, one BDAB^2−^ ligand, half of one BPD molecule, and one coordinated water molecule. As illustrated in Figure 5, atom Zn1 in 3 adopted a slightly disordered tetrahedral coordination geometry surrounded by two carboxylate oxygen atoms (O2, O4#1) from two adjacent BDAB^2−^ ligands, one nitrogen atom (N3) from the BPD ligand and one coordinated water molecule (O5). The connection between Zn^2+^ cations and the carboxylate groups of BDAB^2−^ ligands afforded 1D coordination chains (Figure 6A), which were further assembled by the coordination between Zn^2+^ cations and the nitrogen atoms of BPD ligands to give 2D coordination networks (Figure 6B). On closer inspection, due to the existence of the large pores in the 2D networks, it could be found that six adjacent 2D networks could interlace with each other to give 6-fold interpenetrated 2D supramolecular layers *via* the π…π, C-H…π interactions (Figures 6C,D; Supplementary Figure S3A). Furthermore, the interpenetrated layers were joined together to generate the final 3D supramolecular architecture by the noncovalent interactions including hydrogen bonds, π…π and C-H…π interactions (Figure 6E; Supplementary Figure S3B).

**FIGURE 7:**
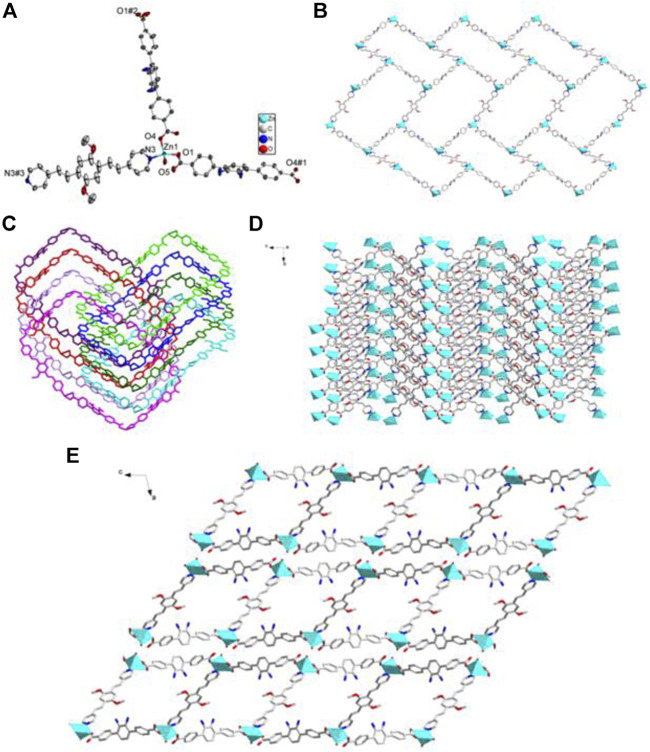
**(A)** Coordination environment of Zn(II) cation in **4** with ellipsoids drawn at 50% probability level. The hydrogen atoms are omitted for clarity. Symmetry codes: #1 *x*,-*y*-1,*z* + 1/2; #2 *x*,-*y*-1,*z*-1/2; #3 -*x*,-*y*+3,-*z*. **(B)** The 2D BDAB^2-^-Zn^2+^-DBPB coordination networks. **(C)** The interlaced mode of the adjacent BDAB^2-^-Zn^2+^-DBPB networks. **(D)** The 2D interpenetrated BDAB^2-^-Zn^2+^-DBPB layers. **(E)** The final 3D supramolecular architecture of 4 with 1D channels along b-axis.

Although a more complicated pyridine ligand DBPB was used instead of BPD to prepare complex 4, the structure of 4 was almost identical to that of 3 and shared the same topological structure with complex 3 (Supplementary Figure S4). Each asymmetric unit of 4 also consisted of one Zn^2+^ cation, one BDAB^2−^ ligand, half of one DBPB molecule, and one coordinated water molecule. Similar to that of 3, the central Zn^2+^ cations in 4 also adopted a distorted tetrahedral geometry (Figure 7A) and connected the organic ligands BDAB^2−^ and DBPB to generate 2D coordination networks (Figure 7B). But differently, due to the much larger size, the pyridine ligand DBPB could allow more 2D BDAB^2−^-Zn^2+^-DBPB coordination networks to interpenetrate with each other to give an 8-fold interpenetrated 2D supramolecular layers (Figures 7C,D; Supplementary Figure S4A). These supramolecular layers further interact with each other to give the final 3D supramolecular frameworks (Figure 7E; Supplementary Figure S4B) via various noncovalent interactions including C-H…π interactions and hydrogen bonds (Supplementary Figure S4C). Furthermore, one more difference between the structure of 3 and 4 was that 1D channels along b-axis could be observed in the framework of 4 (Figure 7E) and the total solvent cavity volume is 22.5% per unit cell calculated by PLATON. (Zou et al., 2010).

### Powder X-ray Diffraction Results and Thermogravimetric Analyses

The PXRD experiments were carried out to confirm whether the crystal structures are truly representative of the bulk materials. The PXRD experimental and computer-simulated patterns of the corresponding complexes are shown in the ESI (Supplementary Figures S5–S8). The experimental data shows that the bulk synthesized materials are the same as the measured single crystals, suggesting the bulk-phase purity of the obtained MOFs. Furthermore, TGA experiments were also carried out in the N_2_ atmosphere from 30 to 500°C to examine the thermal stability of 1–4 and the results were depicted in Supplementary Figures S9–S12. Complex **1** showed a weight loss of 3.6 % from 30 to 260°C, suggesting the release of the coordinated water molecules (calcd 3.92 %) and their structure began to collapse at 400 °C. Complex 2 shows a weight loss of 19 % before 250°C, which corresponds to the release of free water and DMF molecules (calcd 19 %), and further weight loss was observed at about 380°C owing to the collapse of the framework of 2. The TGA curve of complex **3** showed that the framework structure began to decomposing at 350°C. Complex **4** displayed a weight loss of 5.4 % before 110°C corresponding to the release of free water molecules (calcd 5.6 %) and then a weight loss of 2.7 % between 110 and 180°C corresponding to the release of coordinated water molecules (calcd 2.8%). Further quick weight losses were observed at 360°C owing to the decomposition of the frameworks of 4.

### Fluorescence Properties and Sensing Capacity

**FIGURE 8:**
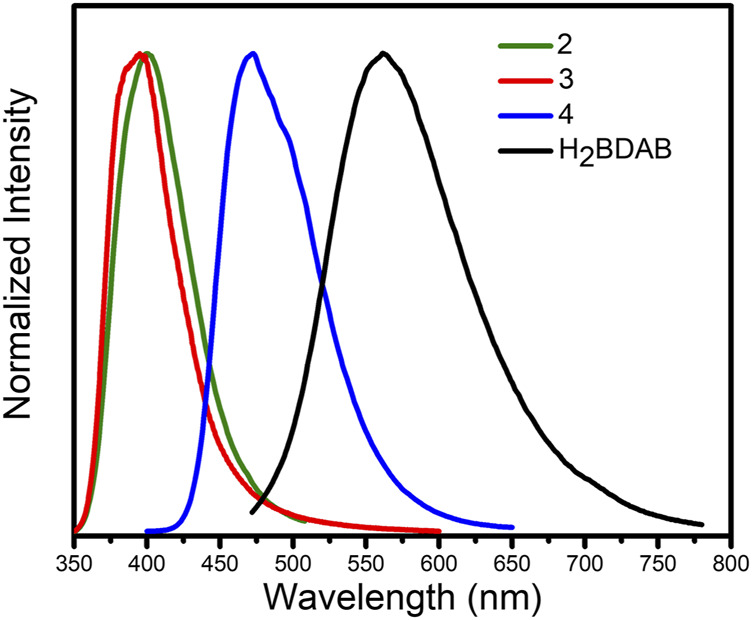
Fluorescence emission spectra of 2–4 and ligand H_2_BDAB.

**FIGURE 9:**
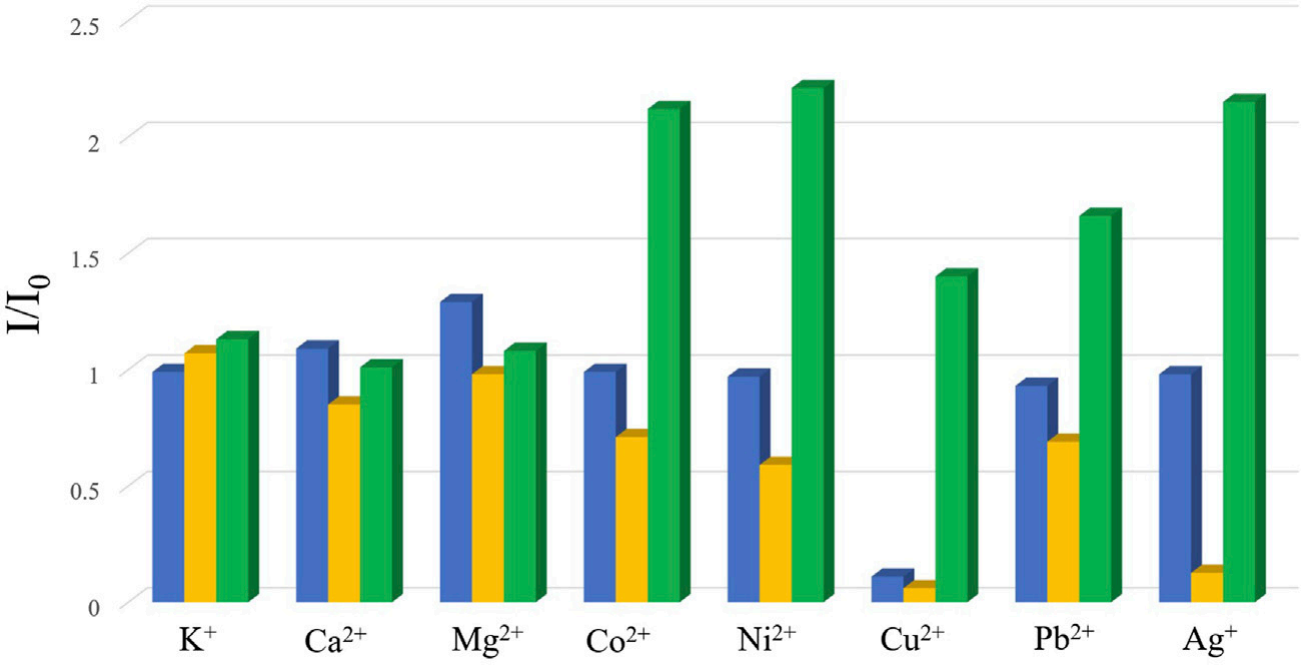
The changes in the emission intensities of 2–4 (blue: 2; yellow: 3; green: 4) upon the addition of the solutions of vairous metal ions.

Previous studies have demonstrated that MOFs containing d^10^-metal ions usually exhibit outstanding fluorescence properties and could function as sensing materials for various substances. On the other hand, in consideration of the existence of channels or uncoordinated amino groups in the structure of complexes 2–4, their fluorescence properties and sensing capability were checked. Thence, the solid-state fluorescence properties of 2–4 were firstly examined at room temperature. As illustrated in Figure 8, the free ligand H_2_BDAB exhibited characteristic emission bands with maxima at 571 nm upon excitation at 356 nm, while the fluorescence emission maxima of 2–4 were observed at 401, 396, and 473 nm upon excitation at 330, 330, and 380 nm, respectively. Compared to the free ligand, apparent blue-shift emissions were observed for 2–4, which may be attributed to the coordination of multi-aromatic ligands to the metal centers. (Nagarkar et al., 2015) Then, the sensing capacities of 2–4 towards common metal ions were checked as well. Before the sensing experiments, the powder samples of 2–4 were fully ground and immersed in DMF to prepare stable suspension (1.0 mg ml^−1^), respectively. Then, the DMF solutions (50 μl, 100 mM) containing different metal ions, including K^+^. Mg^2+^, Ca^2+^, Co^2+^, Ni^2+^, Cu^2+^, Pb^2+^, and Ag^+^, were added into the DMF suspension of 2–4. The changes in the fluorescence emission intensities were recorded (Supplementary Figures S13–S15) and the results were depicted in Figure 9. It could be found that the existence of Cu^2+^ could cause obvious reduction in the fluorescence intensity of 2 while there was no significant change for other metal ions. As for 3, the addition of Cu^2+^ and Ag^+^ both lead to the quenching of the fluorescence quenching and the addition of other metal ions only caused slight or moderate change in the emission intensities of 3. The fluorescence emissions of 4 were either almost unchanged or enhanced and no specific response was observed for metal ions. Therefore, complex 2 may function as a fluorescent sensor for Cu^2+^ and complex 3 could detect Cu^2+^ and Ag^+^ *via* fluorescence quenching effect.

**FIGURE 10:**
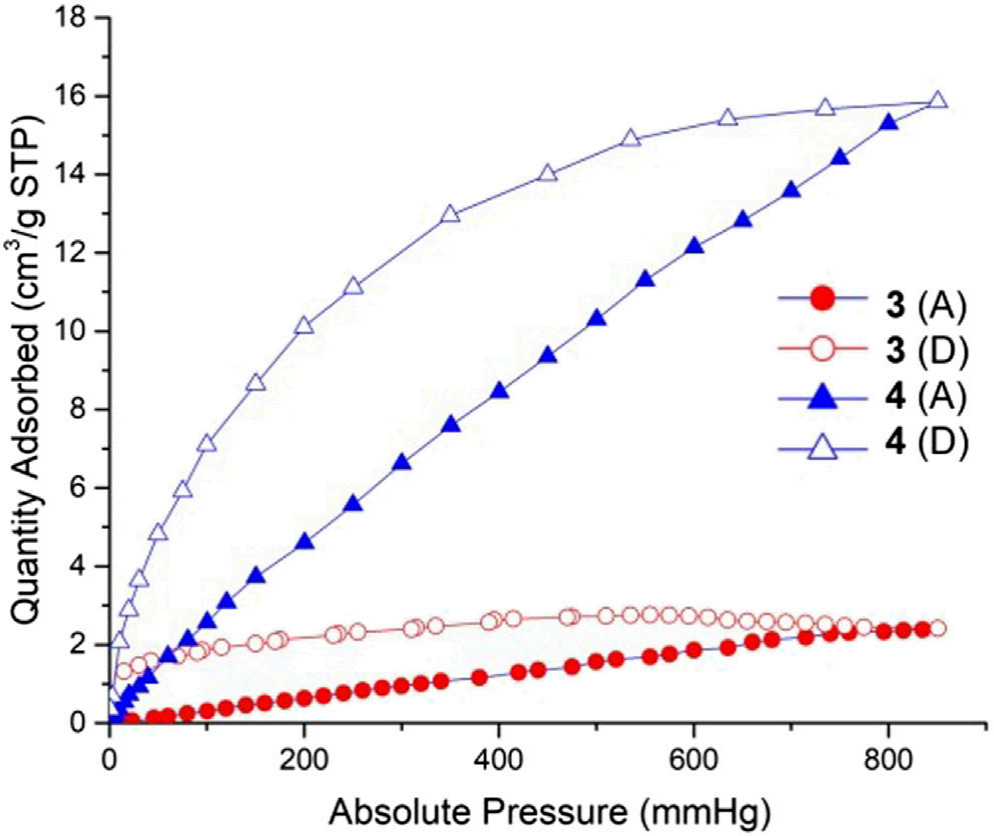
CO_2_ sorption isotherms for complexes 3 and 4 at 298 K. Filled symbols: adsorption; empty symbols: desorption. Red points and lines: complex 3; Blue points and lines: complex 4.

### Carbon Dioxide Adsorption Properties

Coordination polymers 3 and 4 can maintain the stability of the frameworks after 10 h heating and activation. We using CO_2_ as the adsorptive gas to measure the sorption properties of these two complexes. The CO_2_ sorption isotherms of these two complexes measured at 298 K are shown in Figure 7. The gas sorption isotherms indicated a CO_2_ uptake of 2.4 cm^3^/g for 3, 15.9 cm^3^/g for 4. Complex 4 exhibits much more adsorption than complex 3 due to its high porosity: 1,455.9 Å^3^ and the accessible volumes 22.5% for 4. It is speculated that the reason for the low porosity of 3 is that the length of the co-ligand in 3 is relatively short, and the double interspersed would serve to occupy more of the free void space within the porous structure, so that the carbon dioxide molecules cannot enter the hole.

## Conclusion

In summary, amino-functionalized dicarboxylate ligands H_2_BCPAB and H_2_BDAB were employed to react with d^10^ metal ions Cd^2+^ and Zn^2+^ to generate four novel MOFs with the formula of {[Cd(BCPAB)(*μ*_2_-H_2_O)]}_*n*_ (1), {[Cd(BDAB)]∙2H_2_O∙DMF}_*n*_ (2), {[Zn(BDAB)(BPD)_0.5_(H_2_O)]∙2H_2_O}_*n*_ (3) and {[Zn(BDAB)(DBPB)_0.5_(H_2_O)]∙2H_2_O}_*n*_ (4) in the absence and presence of auxiliary pyridyl ligands. Complexes 1 and 2 are 3D frameworks with point symbol of {4.6^2^}{4.6^7^.8^2^} and {4^2^.8^4^}, respectively. Complexes 3 and 4 are generally isostructural and have the similar 3D supramolecular frameworks constructed from 6-fold to 8-fold 2D interpenetrated coordination layers. The fluorescence properties of 2–4 were studied and their capacity as fluorescent sensors for metal ions were explored as well. In addition, the adsorption properties of 3 and 4 for CO_2_ were investigated. The sensing experiments suggested that complex 2 could detect Cu^2+^ and complex 3 could act as a sensor for Cu^2+^ and Ag^+^ *via* quenching effect.

## Data Availability

The original contributions presented in the study are included in the article/Supplementary Material, further inquiries can be directed to the corresponding authors.
