# Supplementary material for: Four Novel d10 Metal-Organic Frameworks Incorporating Amino-Functionalized Carboxylate Ligands: Synthesis, Structures, and Fluorescence Properties
Source: Front Chem. 2021 Aug 30;9:708314. doi: 10.3389/fchem.2021.708314 (PMC8435610; doi:10.3389/fchem.2021.708314)
Supplement: Supplementary file 1 [file DataSheet1.doc]

SUPPLEMENTARY MATERIALS

Four novel d^10^ Metal-Organic Frameworks Incorporating Amino-Functionalized Carboxylate Ligands: Synthesis, Structures, and Fluorescence Properties

Wang Xie^1^, Jie Wu^1^, Xiaochun Hang^1^, Honghai Zhang^1^, Kang shen^1,2^* and Zhoulu Wang^1^*

1 Key Laboratory of Flexible Electronics & Institute of Advanced Materials, Jiangsu National Synergistic Innovation Center for Advanced Materials, School of Energy Science and Engineering， Nanjing Tech University, 30 Puzhu South Road, Nanjing, 211816, China.

2 State Key Laboratory of Coordination Chemistry, Nanjing University.

*Correspondence: Kang shen, [iamkangshen@njtech.edu.cn](mailto:iamkangshen@njtech.edu.cn); Zhoulu Wang, 736481351@qq.com.

**Crystal Structure Determination:** Single crystal X-ray data of complexes **1**-**4** were collected at 293 K on a Bruker SMART APEX CCD diffractometer using graphite monochromated Mo Kα radiation (*λ* = 0.71073 Å). The linear absorption coefficients, scattering factors for the atoms, and the anomalous dispersion corrections were referred to from the International Tables for X-ray Crystallography.^1^ The structures were solved by direct methods, and the non-hydrogen atoms were located from the trial structure and then refined anisotropically using full-matrix least-squares procedures based on *F*^2^ values using SHELXTL (version 6.10) crystallographic software.^2^ The H-atoms attached to carbon atoms were positioned geometrically and treated as riding atoms using SHELXL default parameters. The crystal and refinement data are collected in Table 1. Selective bond distances and angles are given in Table S1 (Supporting Information). The topological analysis and some diagrams were produced using the TOPOS program.^3^

Table S1. Selected Bond Lengths (Å) and Angles (deg) for Complexes **1**-**4**.

| **1** | | | |
| --- | --- | --- | --- |
| Cd(1)-O(2) | 2.272(5) | O(2)-Cd(1)-O(5) | 85.20(17) |
| Cd(1)-O(3)#1 | 2.282(5) | O(3)#1-Cd(1)-O(5) | 83.80(16) |
| Cd(1)-O(5) | 2.391(4) | O(2)-Cd(1)-O(5)#2 | 84.39(17) |
| Cd(1)-O(5)#2 | 2.428(4) | O(3)#1-Cd(1)-O(5)#2 | 82.97(16) |
| Cd(1)-N(1)#3 | 2.425(6) | O(5)-Cd(1)-O(5)#2 | 80.36(9) |
| Cd(1)-O(1) | 2.443(5) | O(2)-Cd(1)-N(1)#3 | 97.36(19) |
| Cd(1)-O(4)#1 | 2.448(5) | O(3)#1-Cd(1)-N(1)#3 | 88.69(18) |
| O(2)-Cd(1)-O(3)#1 | 164.46(18) | O(5)-Cd(1)-N(1)#3 | 157.30(19) |
| O(5)#2-Cd(1)-N(1)#3 | 77.47(17) | O(2)-Cd(1)-O(1) | 55.36(18) |
| O(3)#1-Cd(1)-O(1) | 135.48(17) | O(5)-Cd(1)-O(1) | 90.19(15) |
| O(5)#2-Cd(1)-O(1) | 139.40(15) | N(1)#3-Cd(1)-O(1) | 109.88(18) |
| O(2)-Cd(1)-O(4)#1 | 135.08(18) | O(3)#1-Cd(1)-O(4)#1 | 55.57(16) |
| O(5)-Cd(1)-O(4)#1 | 88.53(15) | O(5)#2-Cd(1)-O(4)#1 | 138.09(15) |
| N(1)#3-Cd(1)-O(4)#1 | 104.80(17) | O(1)-Cd(1)-O(4)#1 | 80.28(15) |
| **2** | | | |
| Cd(1)-N(1)#2 | 2.279(10) | N(1)#3-Cd(1)-C(1)#1 | 96.4(3) |
| Cd(1)-N(1)#3 | 2.279(10) | N(1)#2-Cd(1)-C(1)#1 | 99.5(4) |
| Cd(1)-O(1)#1 | 2.398(8) | N(1)#2-Cd(1)-N(1)#3 | 141.1(5) |
| Cd(1)-O(1) | 2.398(8) | N(1)#3-Cd(1)-O(1)#1 | 92.3(3) |
| Cd(1)-O(2)#1 | 2.363(7) | N(1)#2-Cd(1)-O(1) | 92.3(3) |
| Cd(1)-O(2) | 2.363(7) | N(1)#2-Cd(1)-O(1)#1 | 117.3(3) |
| O(2)-Cd(1)-O(1)#1 | 127.0(3) | N(1)#3-Cd(1)-O(1) | 117.3(3) |
| O(2)-Cd(1)-O(1) | 55.5(3) | N(1)#3-Cd(1)-O(2)#1 | 97.2(3) |
| N(1)#2-Cd(1)-O(1)#1 | 117.3(3) | N(1)#2-Cd(1)-O(2)#1 | 81.9(3) |
| N(1)#3-Cd(1)-O(2) | 81.9(3) | N(1)#2-Cd(1)-O(2) | 97.2(3) |
| **3** | | | |
| Zn(01)-O(2) | 1.915(3) |  |  |
| Zn(01)-O(5) | 1.941(5) | O(2)-Zn(01)-N(3) | 127.99(17) |
| Zn(01)-O(4)#1 | 1.951(3) | O(5)-Zn(01)-N(3) | 99.5(2) |
| Zn(01)-N(3) | 2.008(4) | O(4)#1-Zn(01)-N(3) | 97.30(15) |
| O(4)-Zn(01)#3 | 1.951(3) | O(2)-Zn(01)-O(4)#1 | 109.52(14) |
| O(2)-Zn(01)-O(5) | 109.4(2) | O(5)-Zn(01)-O(4)#1 | 112.5(2) |
| **4** | | | |
| Zn(1)-N(3) | 2.012(3) | O(1)-Zn(1)-O(4) | 109.32(11) |
| Zn(1)-O(1) | 1.907(2) | O(1)-Zn(1)-O(5) | 103.87(11) |
| Zn(1)-O(4) | 1.948(2) | O(4)-Zn(1)-N(3) | 96.88(11) |
| Zn(1)-O(5) | 1.956(3) | O(4)-Zn(1)-O(5) | 113.96(12) |
| O(1)-Zn(1)-N(3) | 130.62(12) | O(5)-Zn(1)-N(3) | 102.34(12) |

Symmetry transformations used to generate equivalent atoms for **1**: #1 x,-y+3/2,z-1/2; #2 -x+1/2,y-1/2,z; #3 -x+1,y-1/2,-z+3/2; #4 x,-y+3/2,z+1/2; #5 -x+1,y+1/2,-z+3/2; #6 -x+1/2,y+1/2,z; for **2**: #1 -y+1/2,-x+1/2,-z-1/2; #2 y+1/2,x-1/2,z-1/2; #3 -x+1,-y,-z; #4 x,-y+1/2,-z+1/2; for **3**: #1 x+1/2,-y+1/2,z+1/2; #2 -x+3/2,-y+7/2,-z+2; #3 x-1/2,-y+1/2,z-1/2; for **4**: #1 x,-y-1,z+1/2; #2 x,-y-1,z-1/2; #3 -x,-y+3,-z.


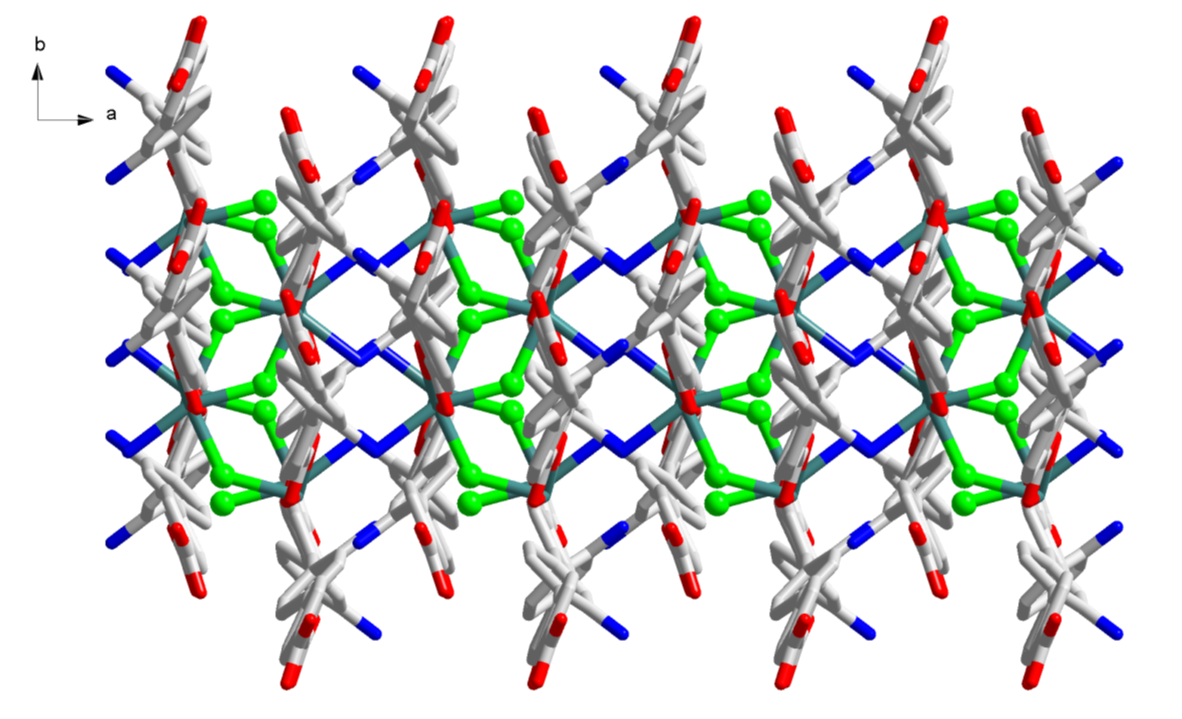

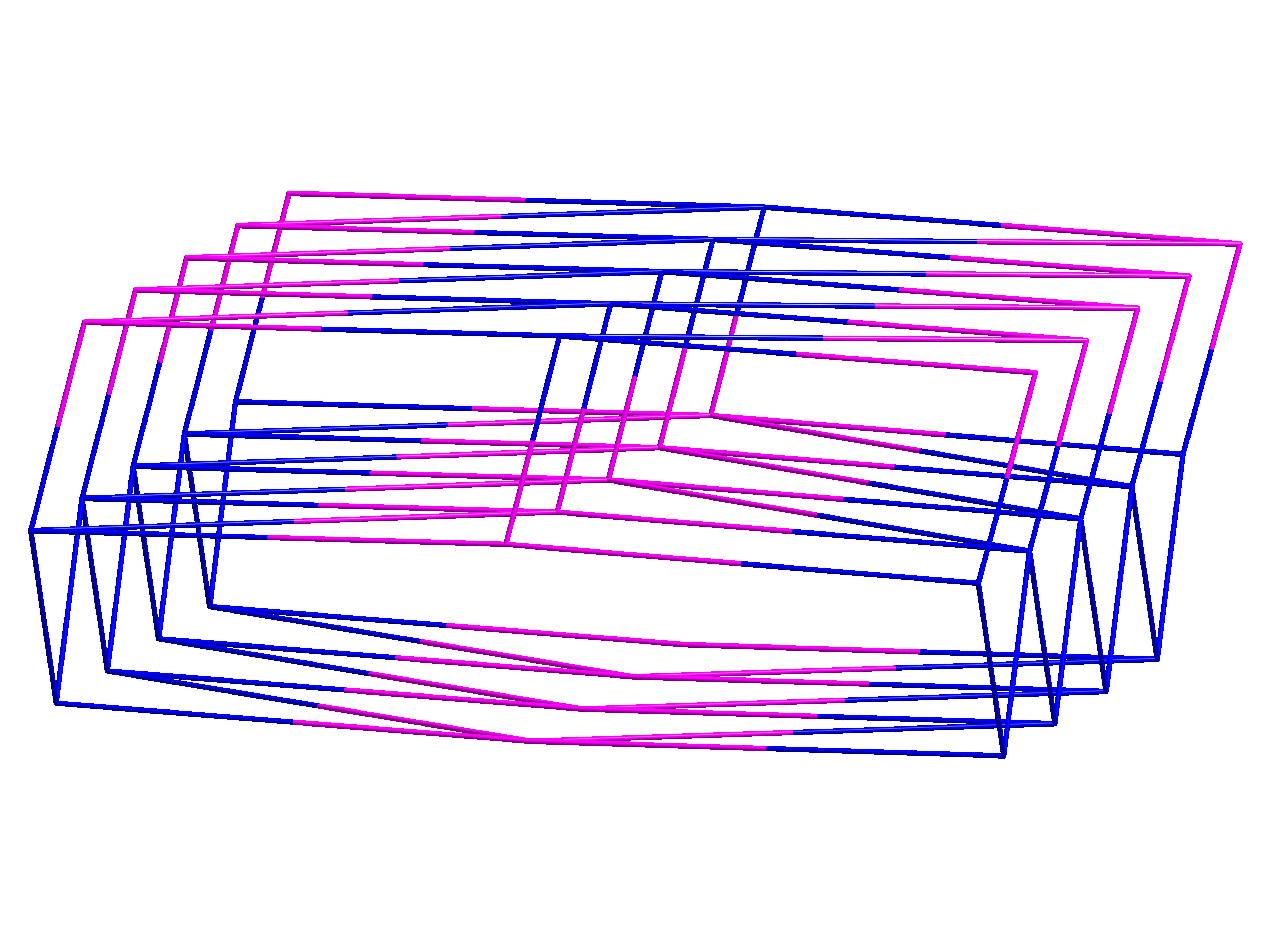


(a) (b)

**Fig. S1** (a) 3D frameworks of 1 along c-axis. (b) Topology of complex **1**.


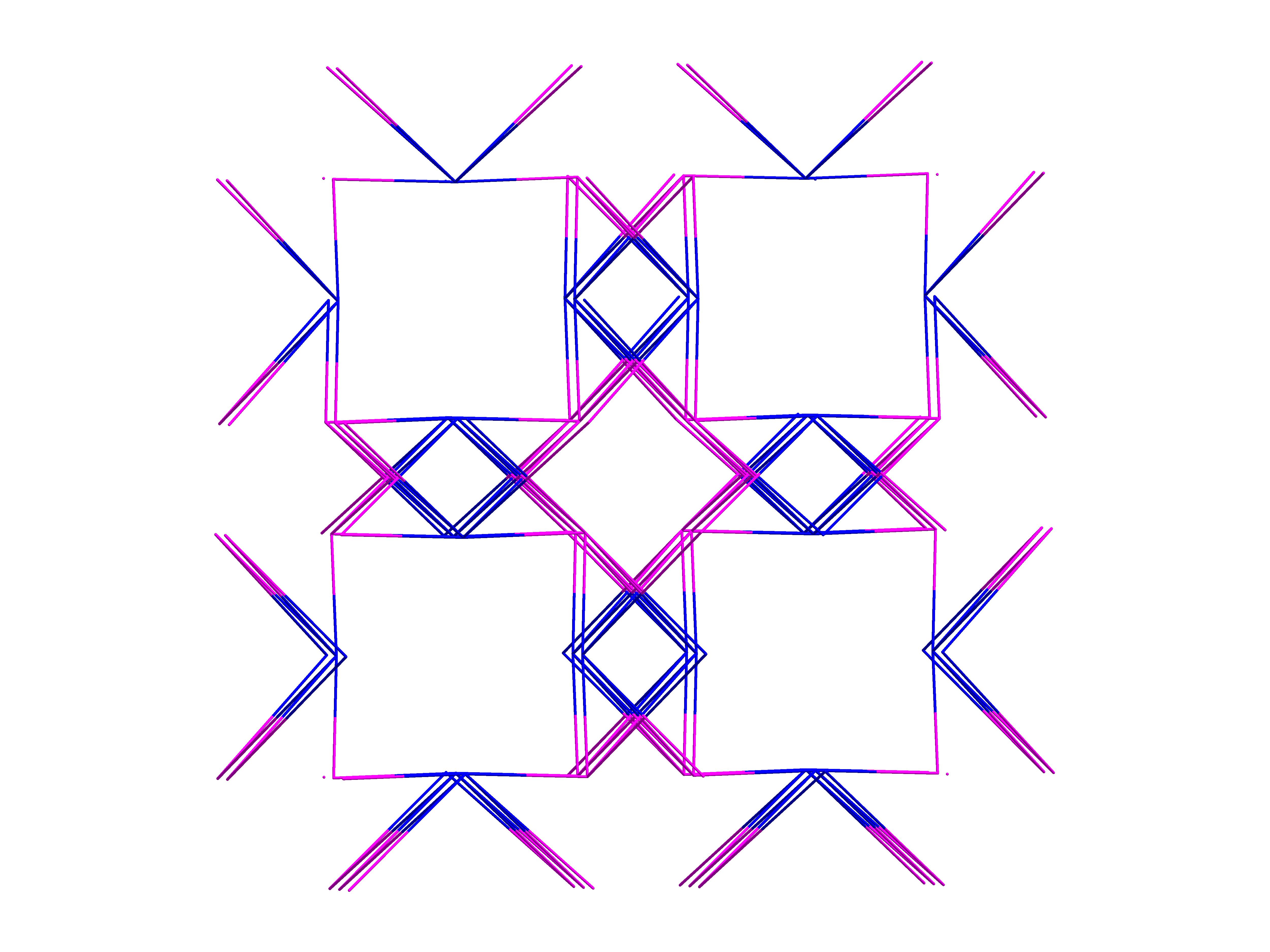


**Fig. S2** Topology of complex **2**.


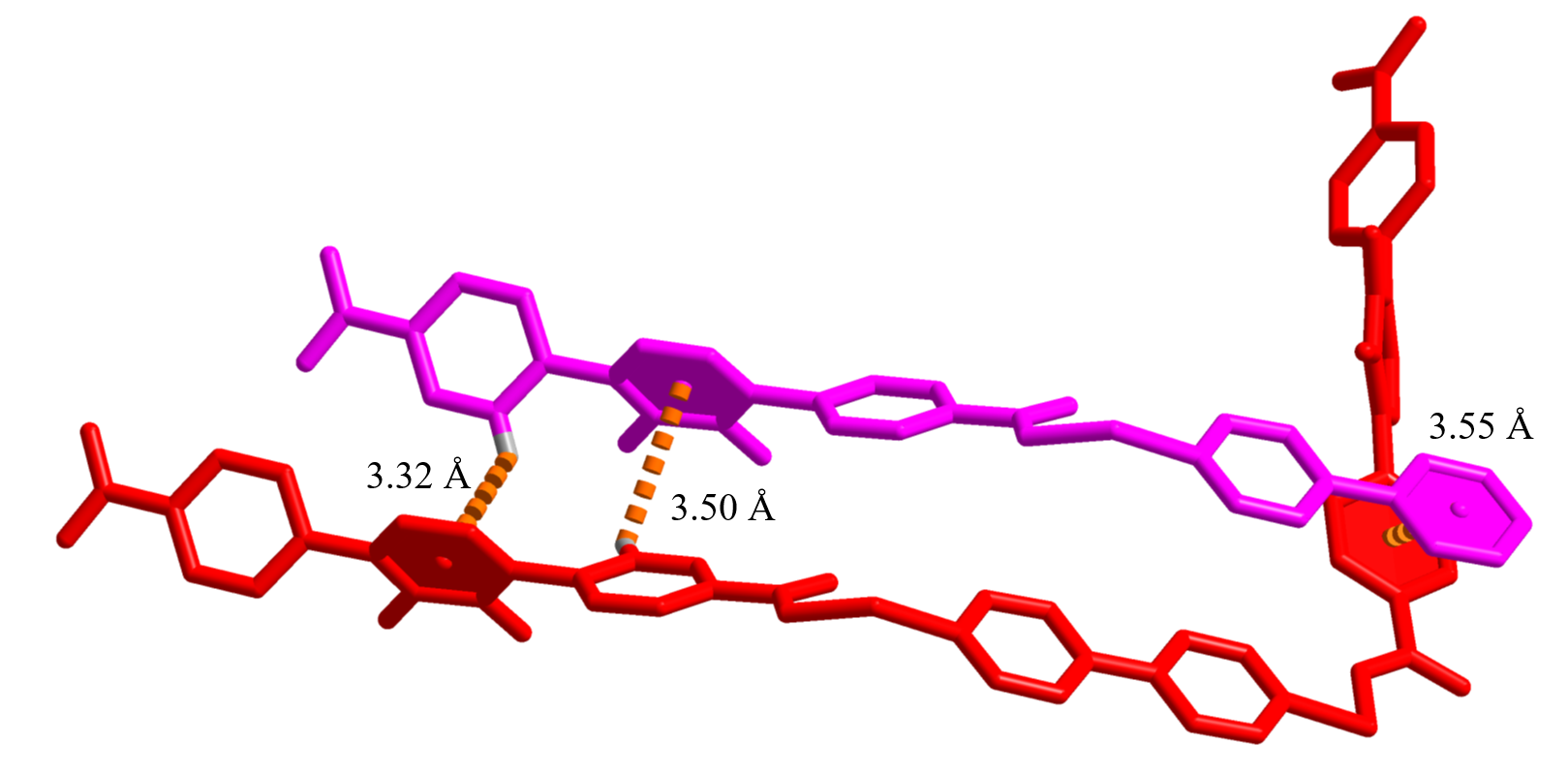

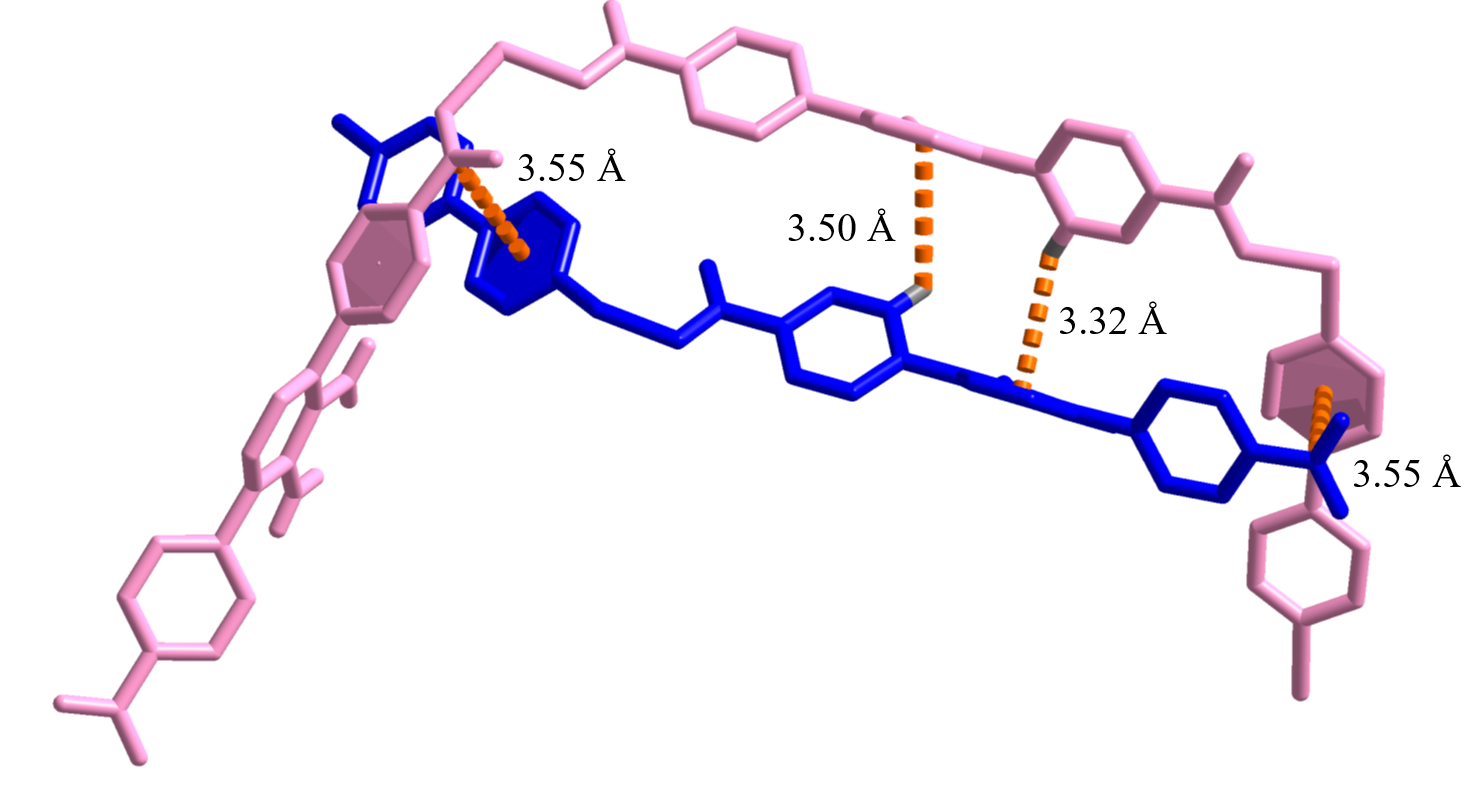


(a)


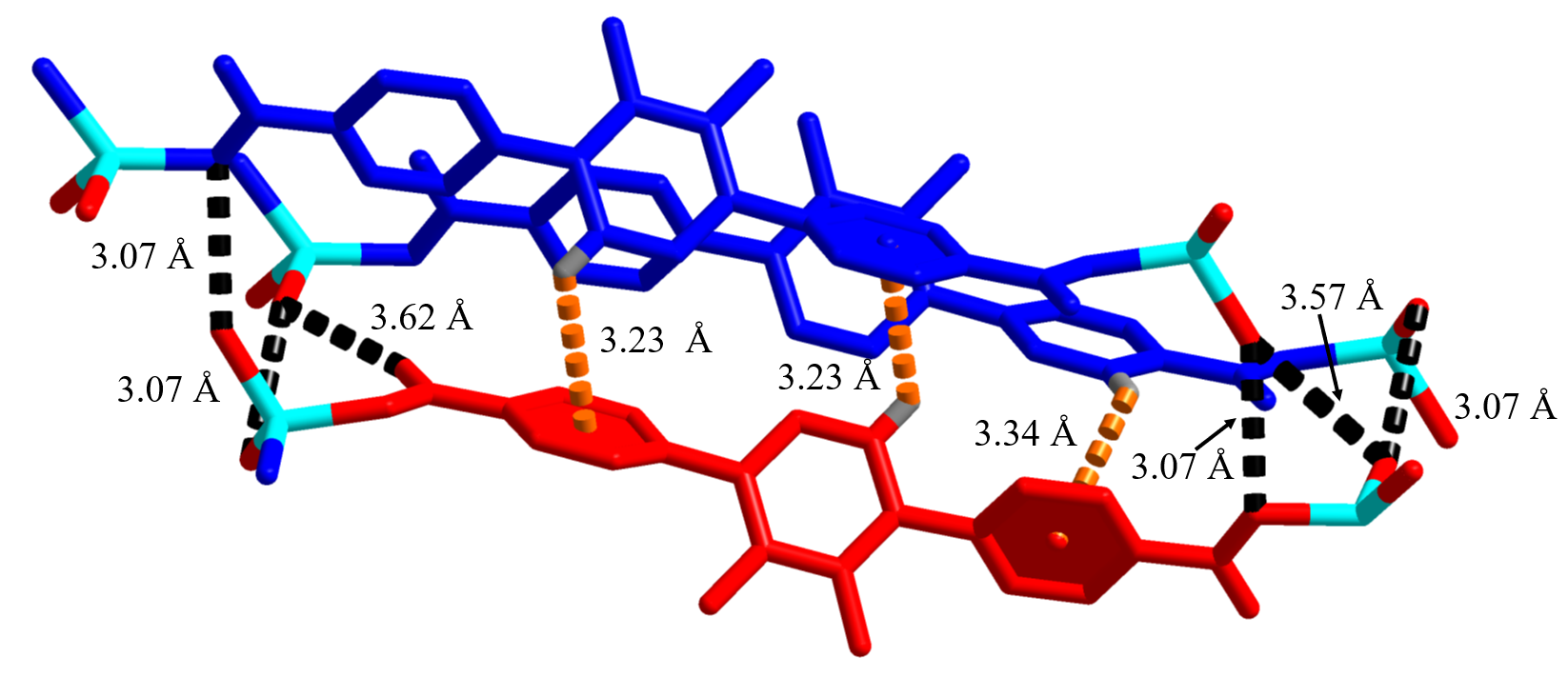


(b)

**Fig. S3** (a) The noncovalent interactions within the 2D interpenetrated BDAB^2-^-Zn^2+^-BPD layers. (b) The noncovalent interactions between neighboring interpentrated layers.


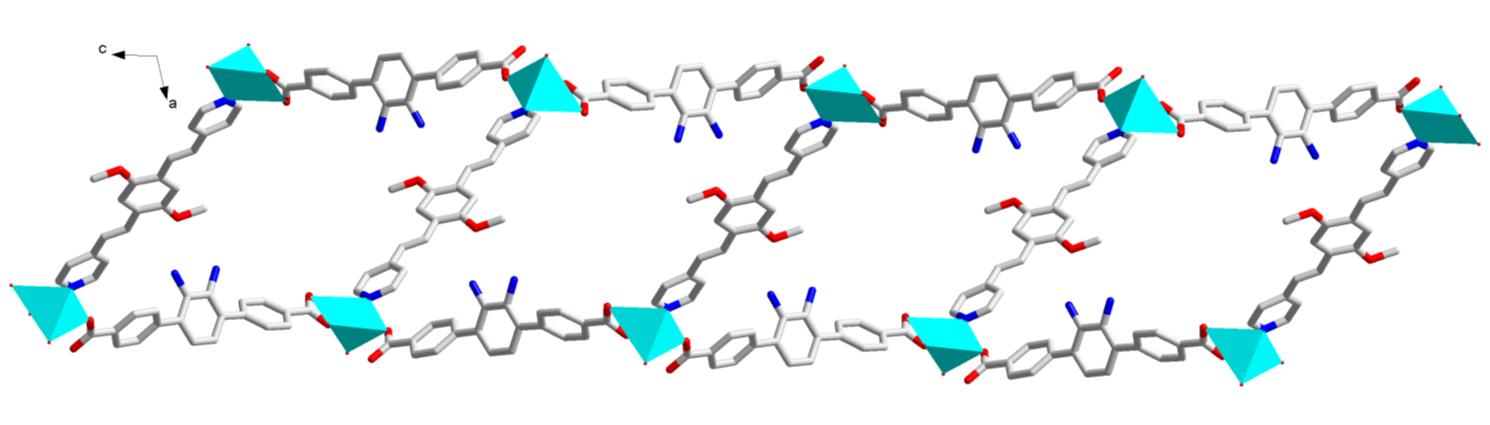


(a)


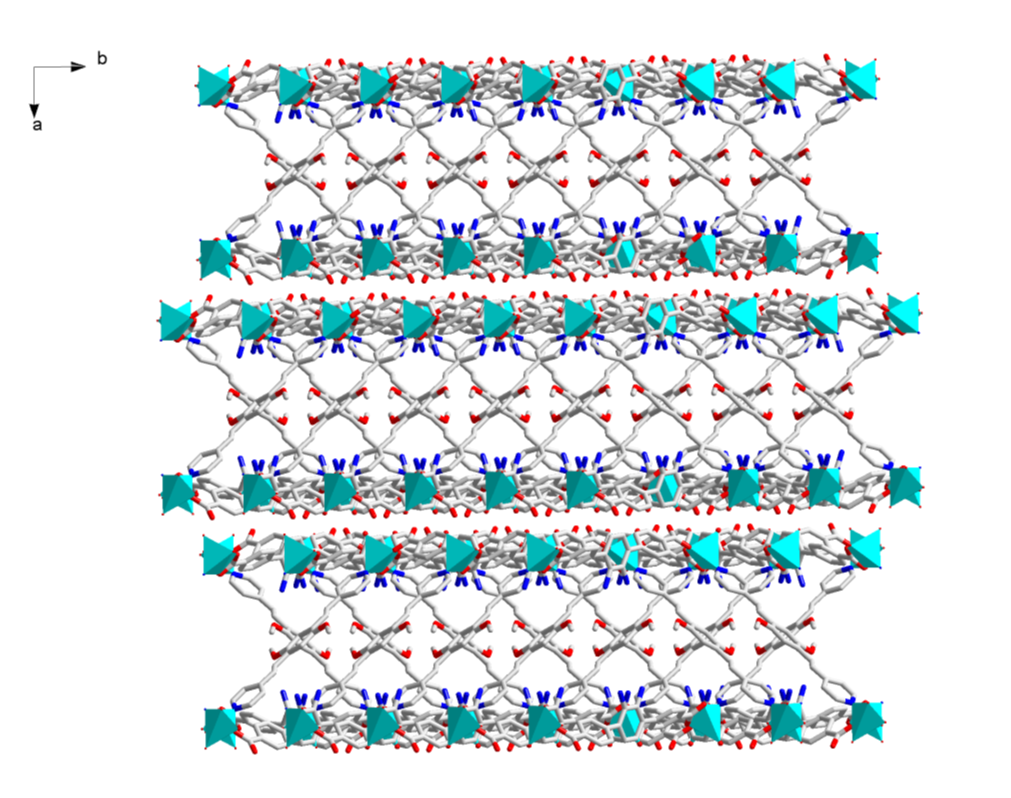


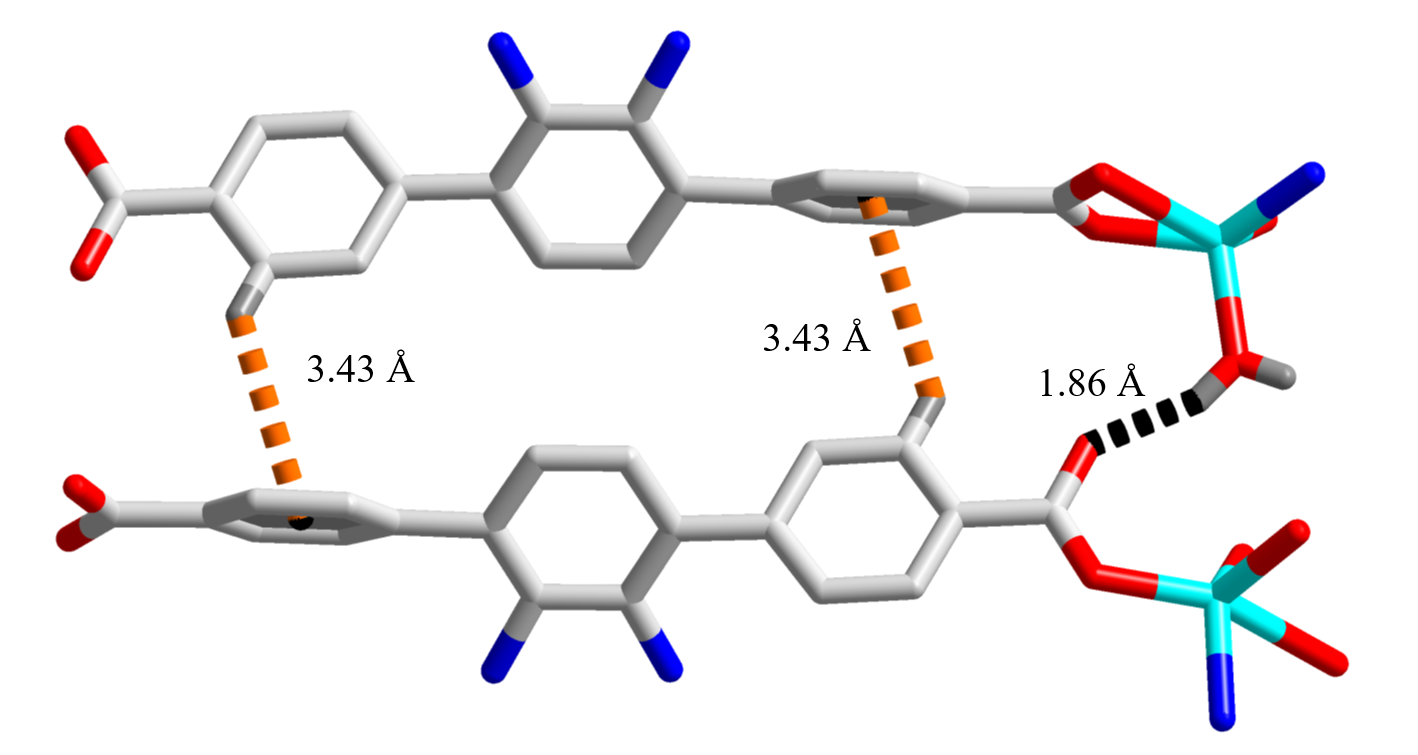


(c)

**Fig. S4** (a) The 2D interpenetrated BDAB^2^--Zn^2+^-DBPB layers along b-axis. (b) The 3D supramolecular framework of **4** along c-axis. (c) The noncovalent interactions between the adjacent layers.


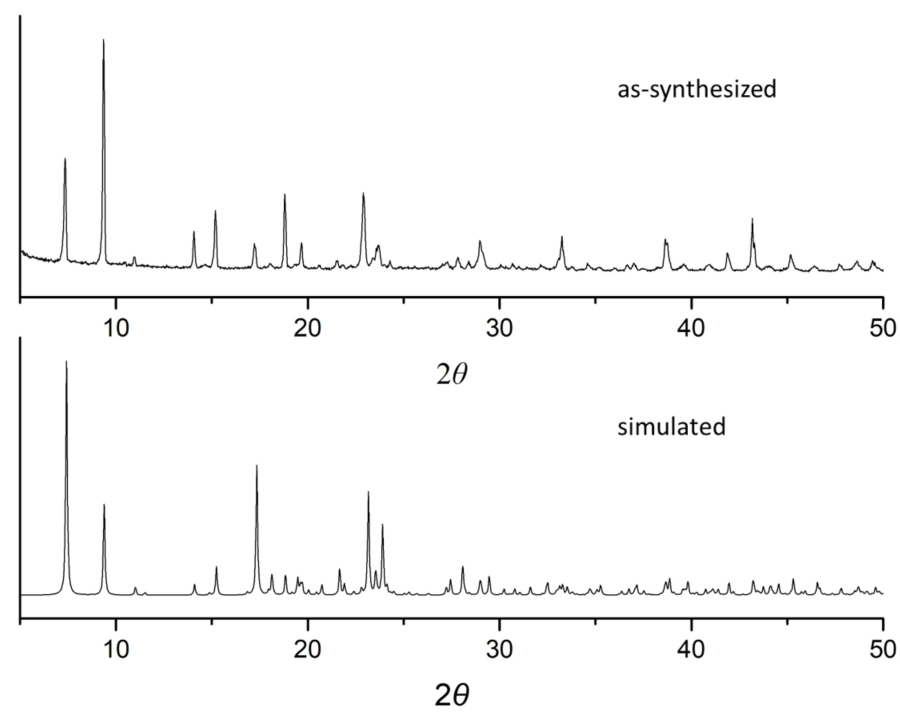


**Fig. S5.** Powder X-ray diffraction patterns of complex **1**.


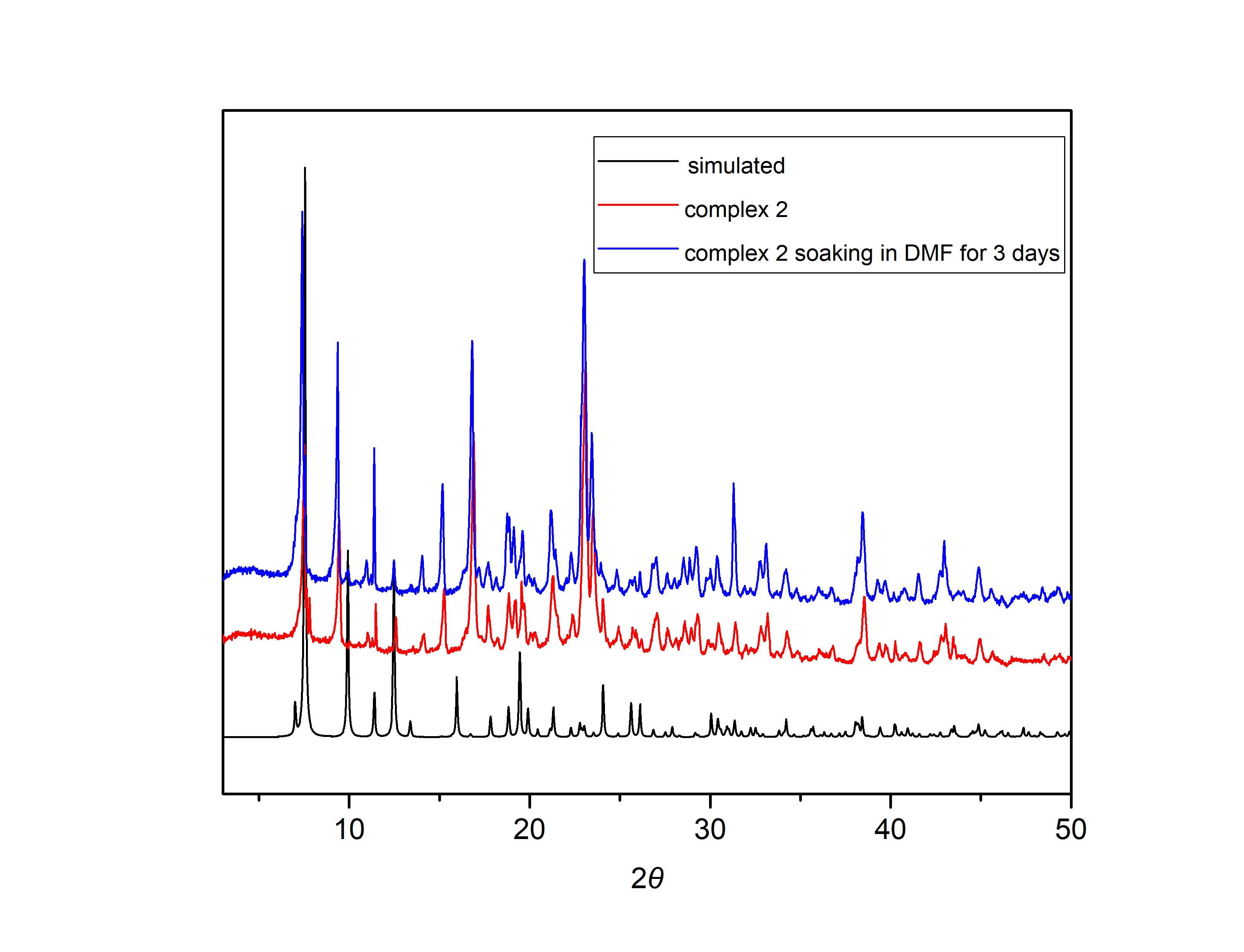


**Fig. S6.** Powder X-ray diffraction patterns of complex **2 complex 2 soaking in water for 3 days.**


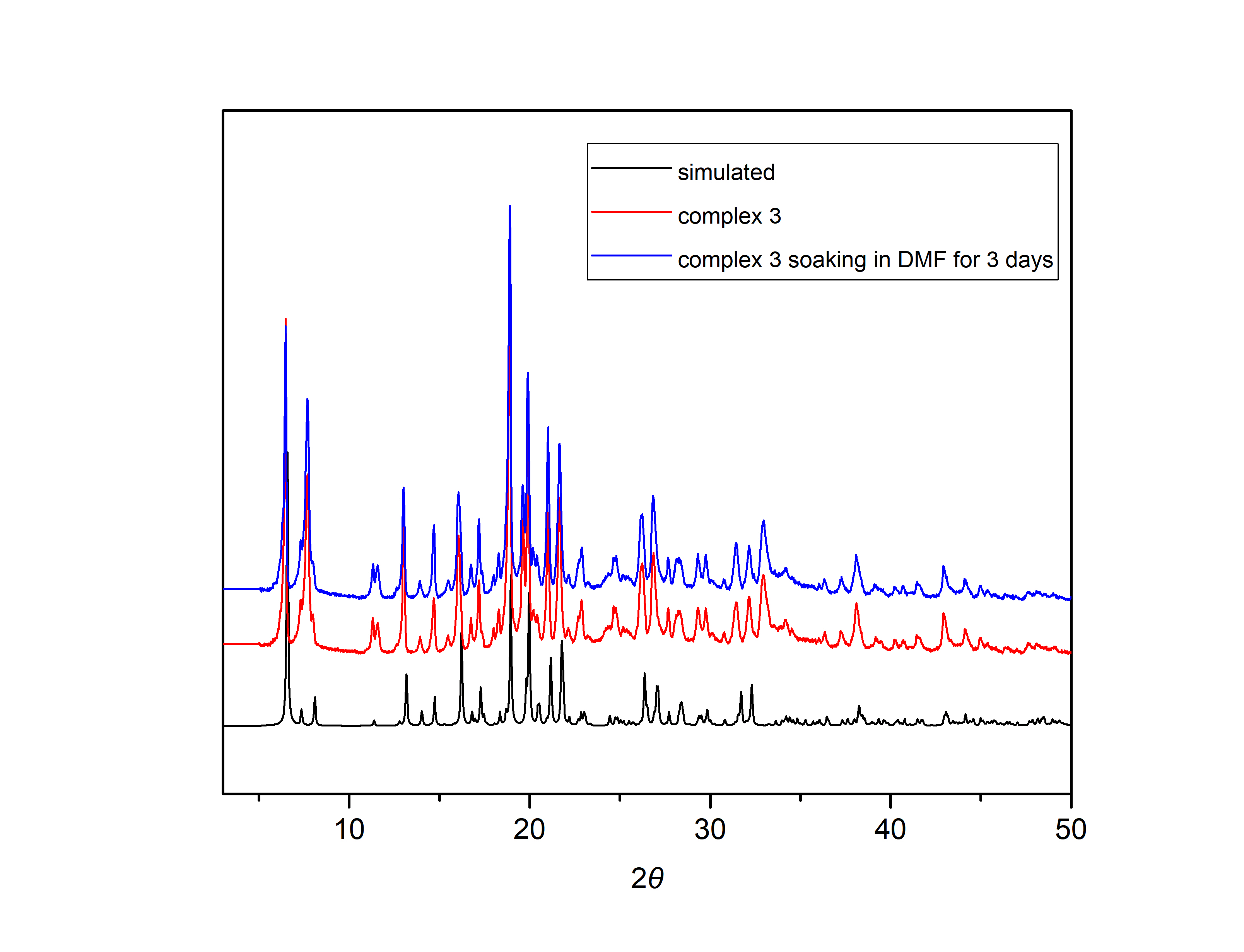


**Fig. S7.** Powder X-ray diffraction patterns of complex **3 complex 3 soaking in water for 3 days.**.


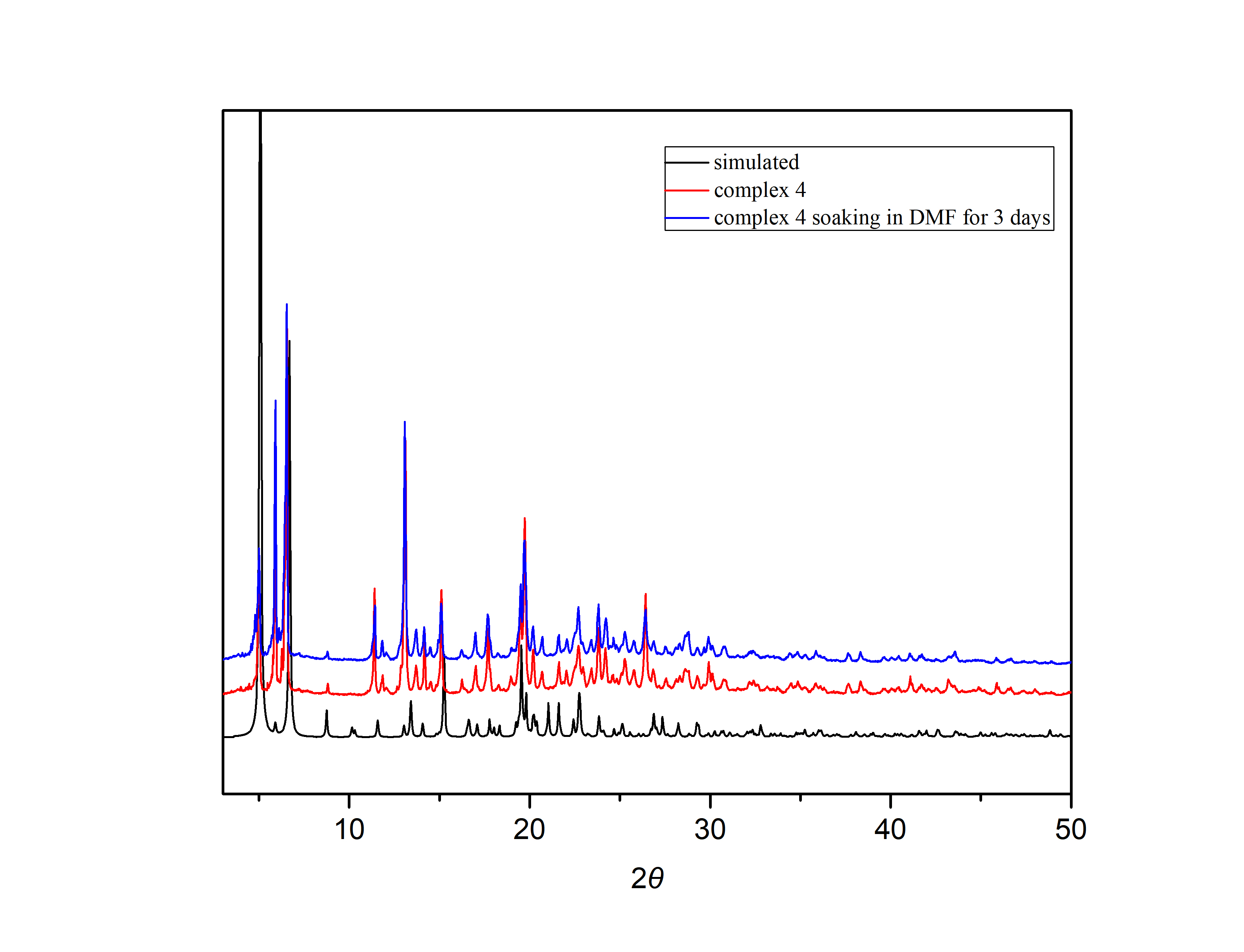


**Fig. S8.** Powder X-ray diffraction patterns of complex **4 complex 4 soaking in water for 3 days.**


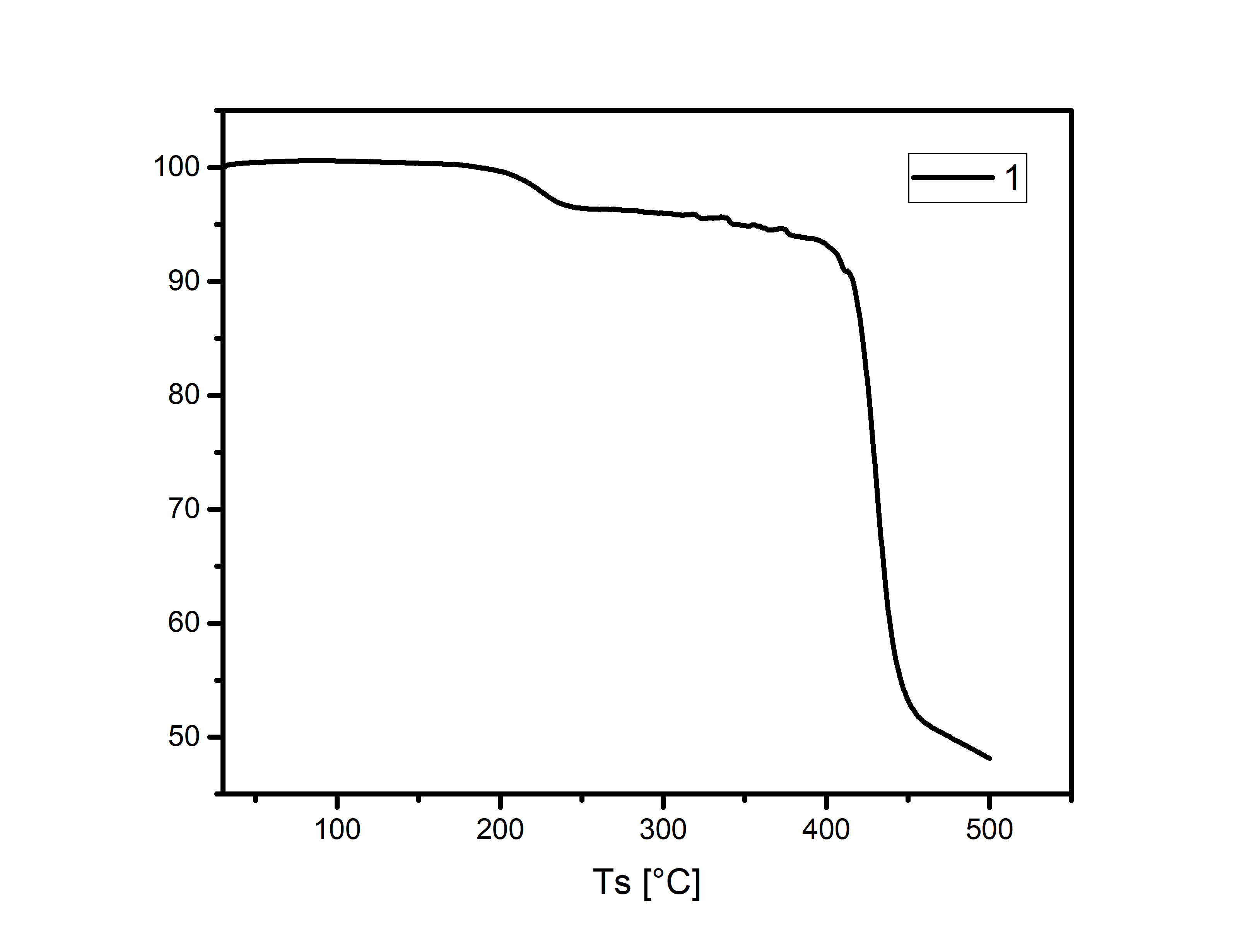


**Fig. S9** TGA data of complex **1**

**
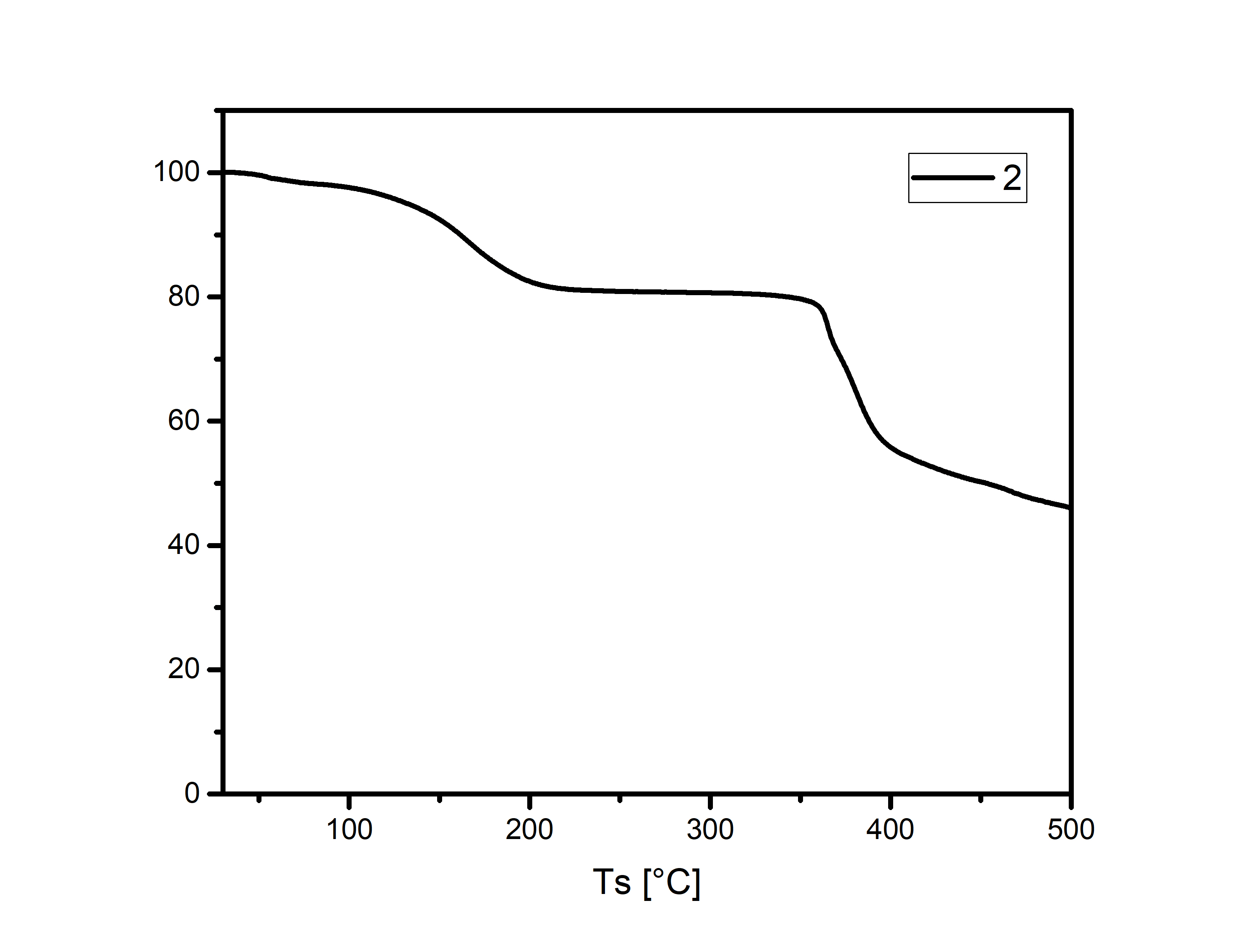
**

**Fig. S10** TGA data of complex **2**.


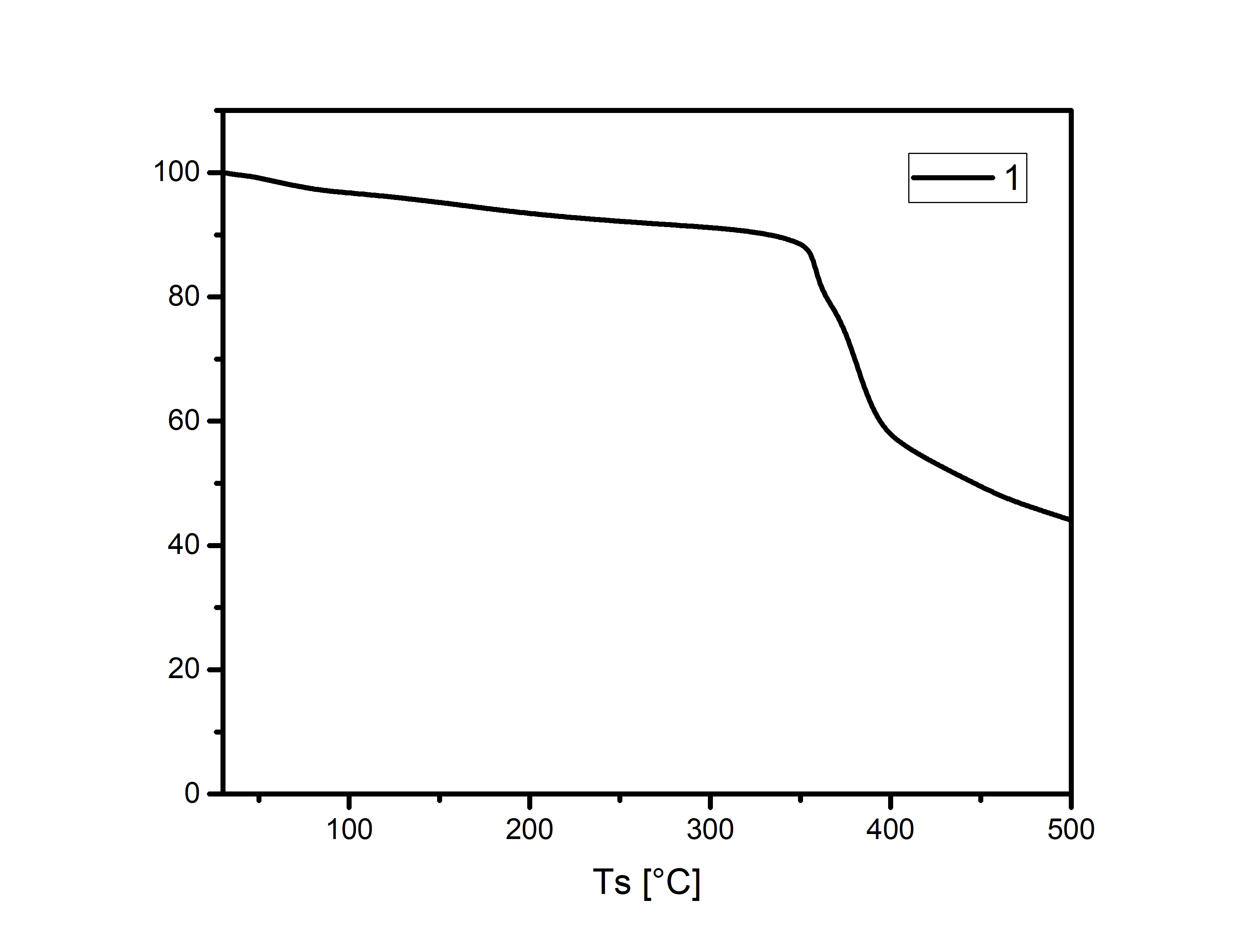


**Fig. S11** TGA data of complex **3**.


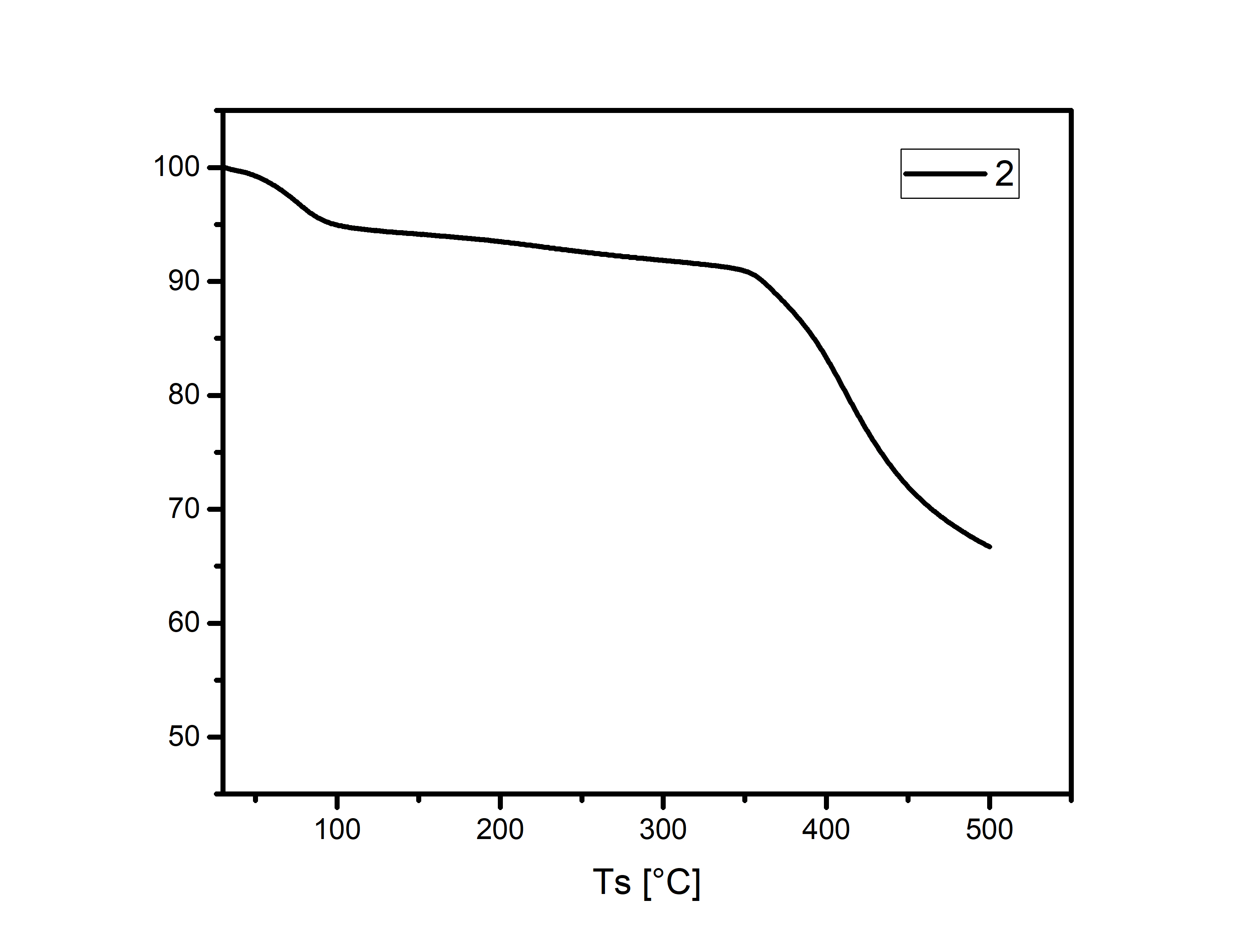


**Fig. S12** TGA data of complex **4**.


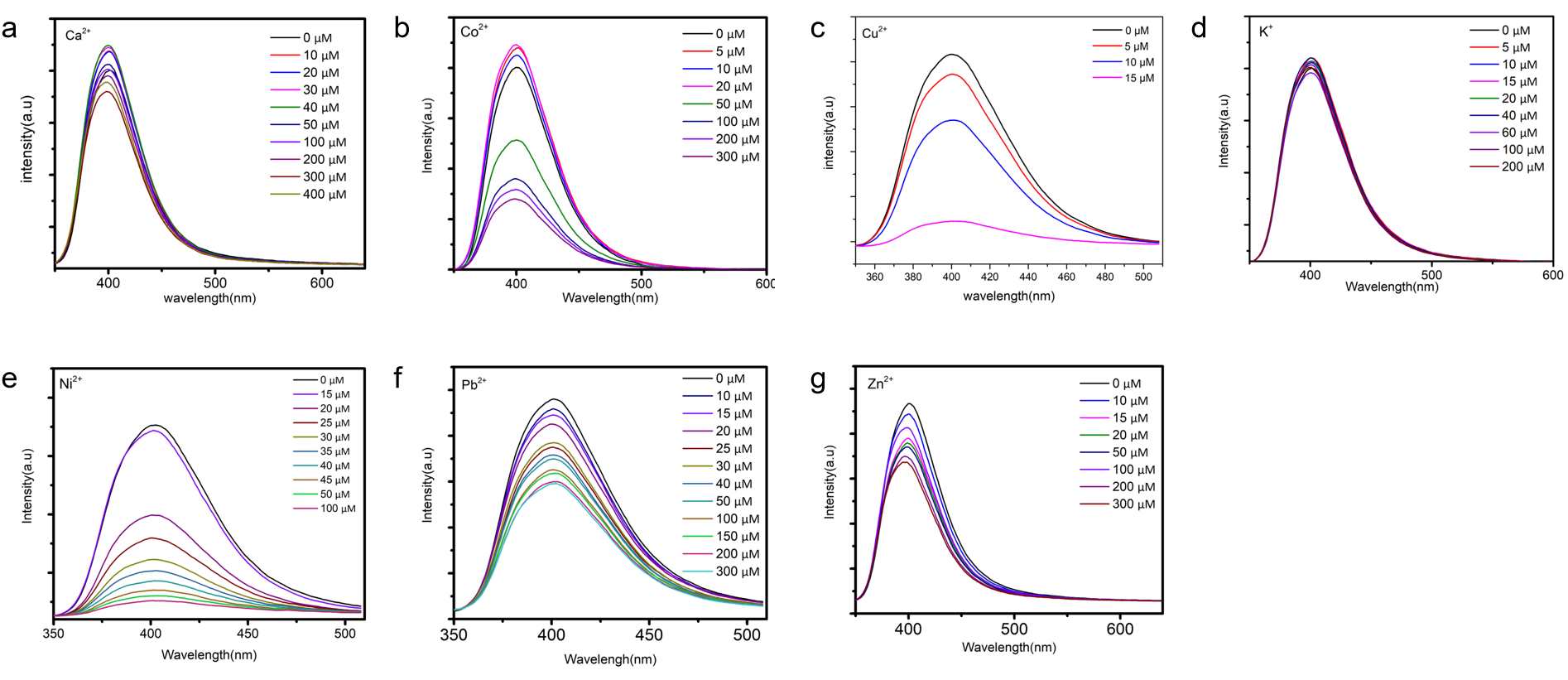


**Fig. S13** Responses of the fluorescence of **2** towards DMF solution of various metal cations.


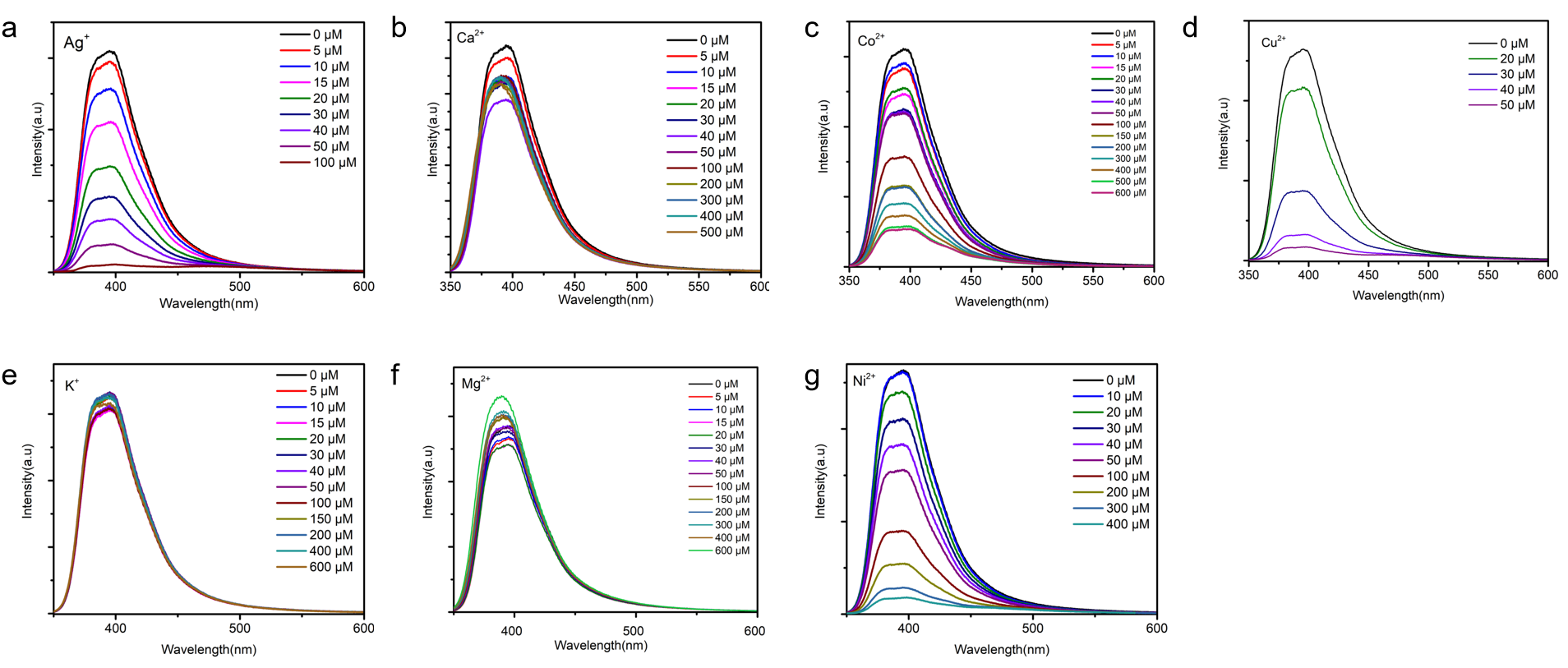


**Fig. S13** Responses of the fluorescence of **3** towards DMF solution of various metal cations.


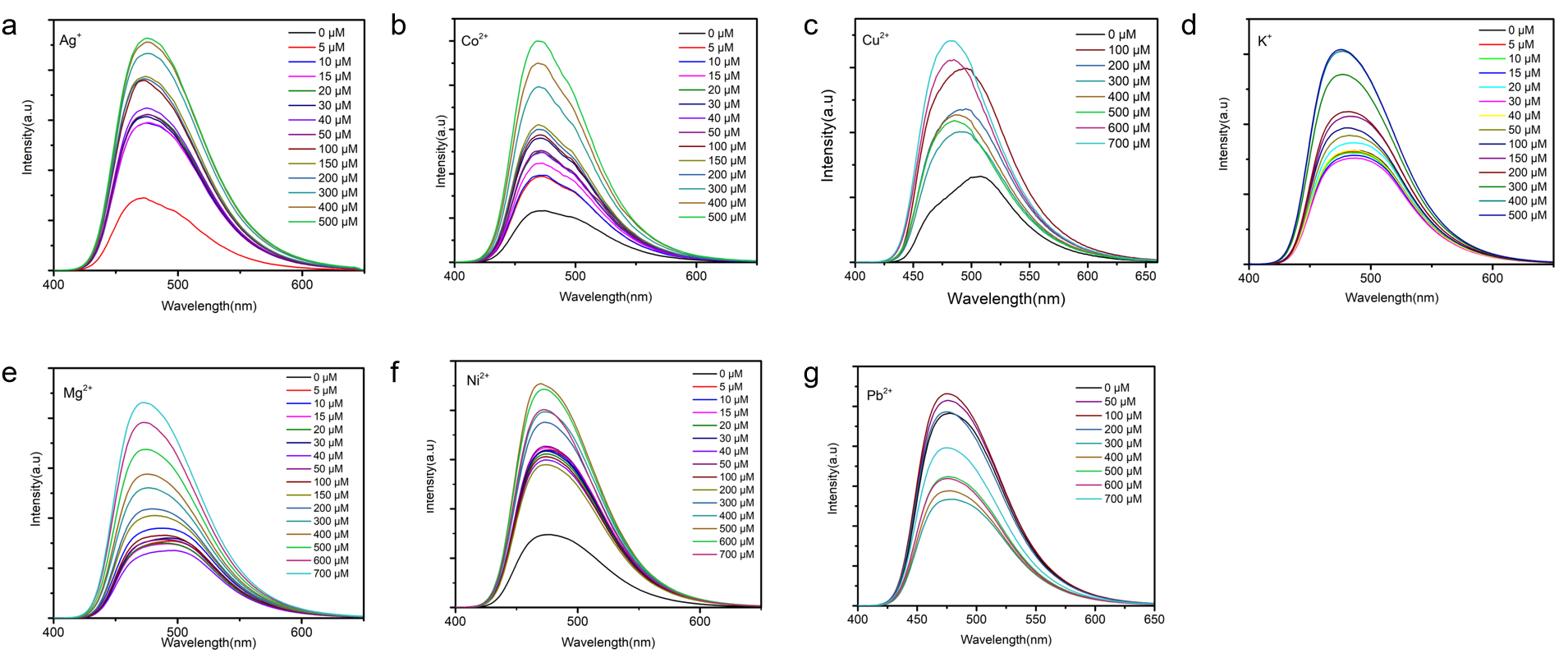


**Fig. S13** Responses of the fluorescence of **4** towards DMF solution of various metal cations.

References

1. International Tables for X-Ray Crystallography; Kynoch Press:Birmingham, England, 1952; Vol. III.

2. SMART (version 5.0), SAINT-Plus (version 6), SHELXTL (version 6.1), and SADABS (version 2.03); Bruker AXS Inc.: Madison, WI.

3. V. A. Blatov, A. P. Shevchenko, V. N. Serezhkin, *J. Appl. Crystallogr.*, 2000, **33**, 1193.
